# Supplementary material for: The formation of the Indo-Pacific montane avifauna
Source: Nat Commun. 2023 Dec 11;14:8215. doi: 10.1038/s41467-023-43964-y (PMC10713610; doi:10.1038/s41467-023-43964-y)
Supplement: Supplementary file 1 — Supplementary Information [file 41467_2023_43964_MOESM1_ESM.pdf]

The formation of the Indo-Pacific montane avifauna

Andrew Hart Reeve, Jonathan David Kennedy, Jose Martin Pujolar, Bent Petersen, Mozes P. K. Blom, Per Alström, Tri Haryoko, Per G. P. Ericson, Martin Irestedt, Johan A. A. Nylander, and Knud Andreas Jønsson

Supplementary Information. Supplementary Note 1, Supplementary Figs. 1–16, Supplementary Tables 1–7.

SUPPLEMENTARY NOTE 1: Detailed results of population studies of montane supercolonizers

Indo-Pacific *Phylloscopus* leaf warblers

Most of the Indo-Pacific *Phylloscopus* leaf warblers, as defined here, form a monophyletic group that is well supported across all analyses (Supplementary Figs. 5–8). This clade includes all populations from the Philippines, Sulawesi, the Moluccas, New Guinea, and islands further east. Support for the broader group, which additionally includes *P. trivirgatus* (Greater and Lesser Sundas) and *P. presbytes* (Lesser Sundas), is slightly more equivocal (pp=0.97 in the supermatrix analysis). This broadly defined Indo-Pacific *Phylloscopus* leaf warbler clade has an estimated divergence date of 3.9 Ma (95% HPD 3.3–4.4 Ma). Estimated clade ages in our dated *Phylloscopus* tree are slightly older than those in previous studies[1,2].

The results of the different phylogenetic analyses vary in some details, but are broadly similar. We first discuss the highly resolved tree generated from the genomic ML analysis. *P. presbytes* is sister to the rest of the clade. *P. trivirgatus* is next to diverge; *P. t. trivirgatus* appears to be paraphyletic with respect to *P. t. parvirostris* (Peninsular Malaysia) and *P. t. kinabaluensis* (n Borneo). Next to diverge is *P. nigrorum* from the Philippines, and then *P. sarasinorum nesophilus* (Sulawesi). The relationships recovered between Sulawesi and Moluccan taxa are less well supported than the rest of the topology (Supplementary Fig. 7 Node A, bootstrap=80; Supplementary Fig. 7 Node B, bootstrap=83). *P. maforensis waterstradti* (Obi and Bacan) is shown to be polyphyletic. Taxa from New Guinea and nearby islands, Kai Besar (Moluccas), the Bismarcks, and the Solomons form a well-supported group. However, *P. maforensis* is paraphyletic with respect to *P. makirensis* (Makira, Solomons), which is embedded deep within the clade as sister to *P. maforensis becki* (Guadalcanal and Malaita, Solomons).

In the supermatrix analysis, the early branching structure of the tree matches that of the genomic ML analysis, though with slightly lower support. However, the supermatrix analysis breaks up the Moluccan clade, recovering Seram (*P. maforensis ceramensis*) and Obi (*P. maforensis waterstradti*) populations as sister to the Philippines clade. Within the Philippines clade, an undescribed population from Mt. Busa (s Mindanao) does not group with other taxa from Mindanao, but rather has a well-supported sister relationship to the Panay population of *P. n. nigrorum*. This relationship was not commented upon in Jones & Kennedy[3], from which the *ND2* sequence derives. The topology recovered for Sulawesi taxa and the remaining Moluccan taxa (*P. nesophilus* and some *P. maforensis* subspecies) is quite different from the genomic ML analysis. This grouping unites taxa from Sulawesi, the Banggai and Sula Islands, and Buru, Bacan and Halmahera (Moluccas) in a well-supported clade. As in the genomic ML analysis, *P. maforensis waterstradti* (Obi and Bacan) is found to be polyphyletic. Taxa from New Guinea and nearby islands, Kai Besar (Moluccas), the Bismarcks, and the Solomons form a fairly well-supported group (pp=0.96), with *P. makirensis* embedded within *P. maforensis* as sister to *P. maforensis becki*. This

analysis also shows that *P. amoenus*, endemic to Kolombangara in the Solomons, is deeply embedded within *P. maforensis*, clustering with other taxa from the Solomons and Bismarcks.

Our analyses reveal that current taxonomic treatments do not accurately reflect the evolution and diversity of the Indo-Pacific leaf warblers. We therefore made a preliminary revision of species limits in order to produce meaningful units for statistical analysis. We applied temporal banding[4] to the supermatrix tree to delimit species, setting the threshold for species-level divergence at 1.42 Ma. This corresponds to the divergence date estimate for the two taxa co-occurring on Kolombangara, *P. amoenus* and *P. maforensis pallescens*; 1.4 Ma is evidently adequate time to establish reproductive isolation between populations in this complex. We delimited 16 species within the complex (Supplementary Fig. 5); the unsampled *P. rotiensis* likely represents an additional species. This treatment is entirely congruent with our genomic ML analysis, except for the inclusion of *P. s. nesophilus* (Sulawesi) in the species that also contains Halmahera and Bacan taxa. We note that further refinement of species limits will ultimately require the incorporation of vocalization and morphological data.

The Indo-Pacific leaf warbler radiation originated some 4 Ma, and appears to have reached as far as the Philippines and New Guinea around the start of the Pleistocene 2.6 Ma (Supplementary Fig. 5; Supplementary Data 4). Diversification surged in the early Pleistocene and continued through most of the epoch, though inter-island diversification seems to have tapered off slightly within the last 200–300 Ka. The complex evolved from Palearctic/Indomalayan ancestors (Supplementary Data 4), and the genomic ML tree indicates a west-to-east stepping-stone expansion out of continental Asia. This includes an ambiguous double colonization of the Lesser Sundas, an expansion into the Philippines, and a broad eastward colonization proceeding through Sulawesi, the Moluccas, New Guinea, and the Bismarcks and Solomons. Indo-Pacific leaf warblers are overwhelmingly montane, but reach the lowlands of Rote and Timor (Lesser Sundas), Numfor and Biak/Supiori (off northern New Guinea), and Mussau (Bismarcks). Except for Timor, these are all small, low-lying islands. The *Phylloscopus* elevational range reconstruction (Supplementary Data 4) and the results of the phylogenetic analyses indicate that these represent 3-4 separate ecological shifts from the ancestral 'montane' state. All members of the complex are essentially sedentary. The *Phylloscopus* migratory behavior reconstruction (Supplementary Data 4) indicates that the last common ancestor (3.9 Ma) was sedentary, but that ancestors from 4.2 Ma and earlier were short-distance migrants.

Snowy-browed Flycatcher *Ficedula hyperythra*

Ficedula hyperythra as currently delimited is paraphyletic, as our results reveal that Damar Flycatcher *F. henrici* is embedded within it (Supplementary Figs. 9–12). All analyses except the mitogenome analysis recover a well-supported sister relationship between *F. henrici* and *F. h. audacis* from the nearby small island of Babar in the Lesser Sundas. All analyses additionally recover a deep divergence between *F. h. annamensis* of southern Vietnam and the rest of *F. hyperythra*, dated at 3.4 Ma (95% HPD 2.4–4.7 Ma) in the supermatrix analysis. While this strongly indicates species-level divergence, we continue to treat subspecies *annamensis* as part of *F. hyperythra* pending an integrative taxonomic assessment. The supermatrix analysis shows that *F. hyperythra* individuals from northern Vietnam are not closely related to *F. h. annamensis*, but a recently discovered population from the Cardamom Mountains of Cambodia is considered to belong to this subspecies[5]. The supermatrix analysis recovers a weakly supported (posterior probability = 0.72) sister relationship between *F. hyperythra* and the Sino-Himalayan *F. tricolor*, with a divergence date of 14 Ma (not shown); this relationship was strongly supported by Moyle et al.[6] and Hooper et al.[7] (2016). The *F. hyperythra* crown group (excluding *F. h. annamensis*) began to diversify at 1.4 Ma (95% HPD 1.1–1.8 Ma).

The supermatrix analysis shows explosive radiation of the group through the Greater Sundas and Wallacean islands beginning at around 1 Ma. It appears that most modern island populations formed during this initial burst, and that inter-island diversification slowed significantly thereafter. Given these temporal diversification dynamics, it is unsurprising that this tree is rather poorly resolved beyond the earliest branches. The genomic ML tree is reasonably well resolved, but receives lower overall support than similar analyses for the other montane supercolonizers. The geographic range reconstruction for the genus *Ficedula* (Supplementary Data 4) strongly indicates that *F. hyperythra* evolved from Palearctic ancestors. The finding that *F. h. annamensis* from south Vietnam is a well-diverged sister to the rest of *F. hyperythra* blurs this picture slightly, but the next branch of the tree represents populations spanning the Palearctic and Indomalayan regions, indicating that this is the ancestral range of the species. From here it appears to have expanded into the Greater Sundas. From the Greater Sundas, the genomic ML analysis indicates that *F. hyperythra* made two separate eastward expansions: one via Sulawesi into the Moluccas, and one through the Lesser Sundas. Two populations on very small islands in the eastern Lesser Sundas occur down to low elevations. On Babar it has been recorded down to 200 masl[8], and on Damar down to 60 masl[9]. Examined together with the *Ficedula* elevational range reconstruction (Supplementary Data 4), the results of the phylogenetic analyses strongly suggest that Babar and Damar populations, and *F. hyperythra* as a whole, evolved from montane ancestors. *F. hyperythra* appears to have evolved from short-distance migrants, based on the *Ficedula* migration reconstructions (Supplementary Data 4) and inclusion of the altitudinal migrant Himalayan population in an early branching clade of *F. hyperythra* (Supplementary Fig 9).

Mountain Tailorbird *Phyllergates cucullatus*
The supermatrix analysis recovered a monophyletic *Phyllergates cucullatus* (Supplementary Figs. 13–16). Divergence from its sister species, *P. heterolaemus* from Mindanao, was estimated at 4.1 Ma (95% HPD 1.8–6.9 Ma). Populations of *P. cucullatus* were estimated to have begun diverging 1.3 Ma (95% HPD 1.0–1.7 Ma). The mainland *P. c. coronatus* may be paraphyletic, based on the highly supported topology from the genomic ML analysis. This analysis groups north and south Vietnamese *P. c. coronatus* populations together as sister to the rest of *P. cucullatus.* Next to diverge are Myanmar and northeast India *P. c. coronatus* populations, which are sister to the remaining taxa. Among Moluccan taxa, all analyses recover a sister relationship between *P. c. dumasi* (Seram) and *P. c. batjanensis* (Bacan), which are in turn sister to *P. c. dumasi* (Buru). These relationships are well supported in all but the supermatrix analysis, indicating that *P. c. dumasi* is paraphyletic.

Phyllergates cucullatus underwent a recent archipelagic radiation beginning c. 1 Ma, similar to *F. hyperythra*. Inter-island diversification seems to have proceeded at a somewhat slower rate than in *F. hyperythra*, occurring from 1 to 0.3 Ma and even more recently. As a consequence of this slower rate of diversification, the supermatrix tree is slightly better supported, and the genomic ML tree is well resolved. The geographic range reconstruction for Cettiidae (Supplementary Data 4) provides somewhat equivocal results as to the ancestral origin of *P. cucullatus*, due to geographic signal from its Philippines-endemic sister species; *Phyllergates* belongs to an otherwise Palearctic/Indomalayan clade. The genomic ML analysis results suggest that *P. cucullatus* evolved from a Palearctic/Indomalayan ancestor, as populations occurring there represent early branches of the tree. From this probable ancestral range, *P. cucullatus* underwent a range expansion similar to that of *F. hyperythra*. It appears to have expanded through the Greater Sundas, and from there to the northern Philippines; east into the Moluccas via Sulawesi; and east into the Lesser Sundas. The elevational range reconstructions coupled with the phylogenetic analyses provide a picture of an entirely montane radiation. The migratory behavior of the species' last common ancestor remains

unclear, but the altitudinal migrant population in northeast India belongs to one of the early branching groups (Supplementary Fig. 13).

SUPPLEMENTARY FIGURES

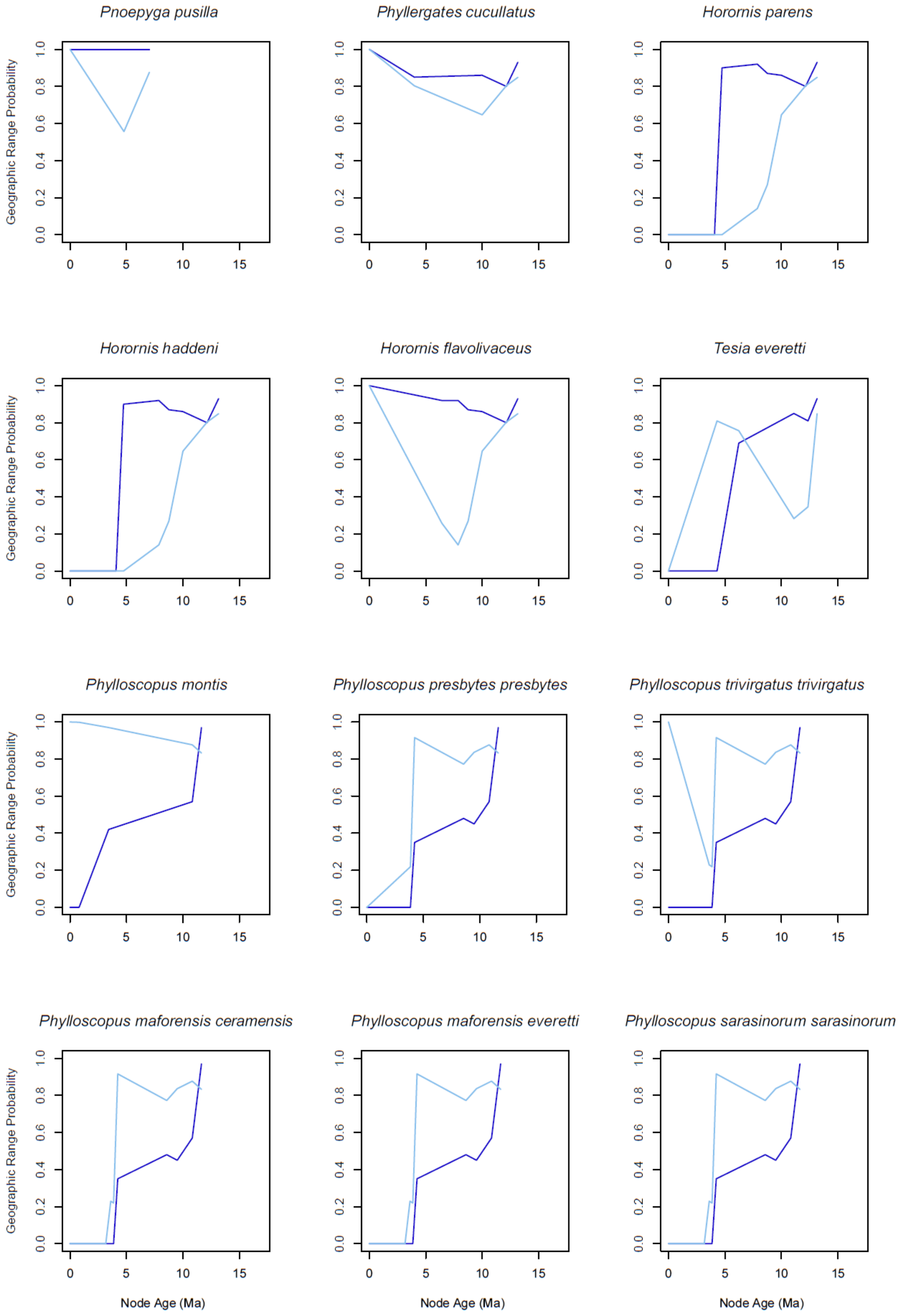

Supplementary Fig. 1a. Ancestral geographic range curves for Eurasian-origin species. Curves link composite region scores for nodes in the ancestral geographic range reconstructions. Dark blue lines connect posterior probabilities of Palearctic occurrence, light blue lines Indomalayan occurrence.

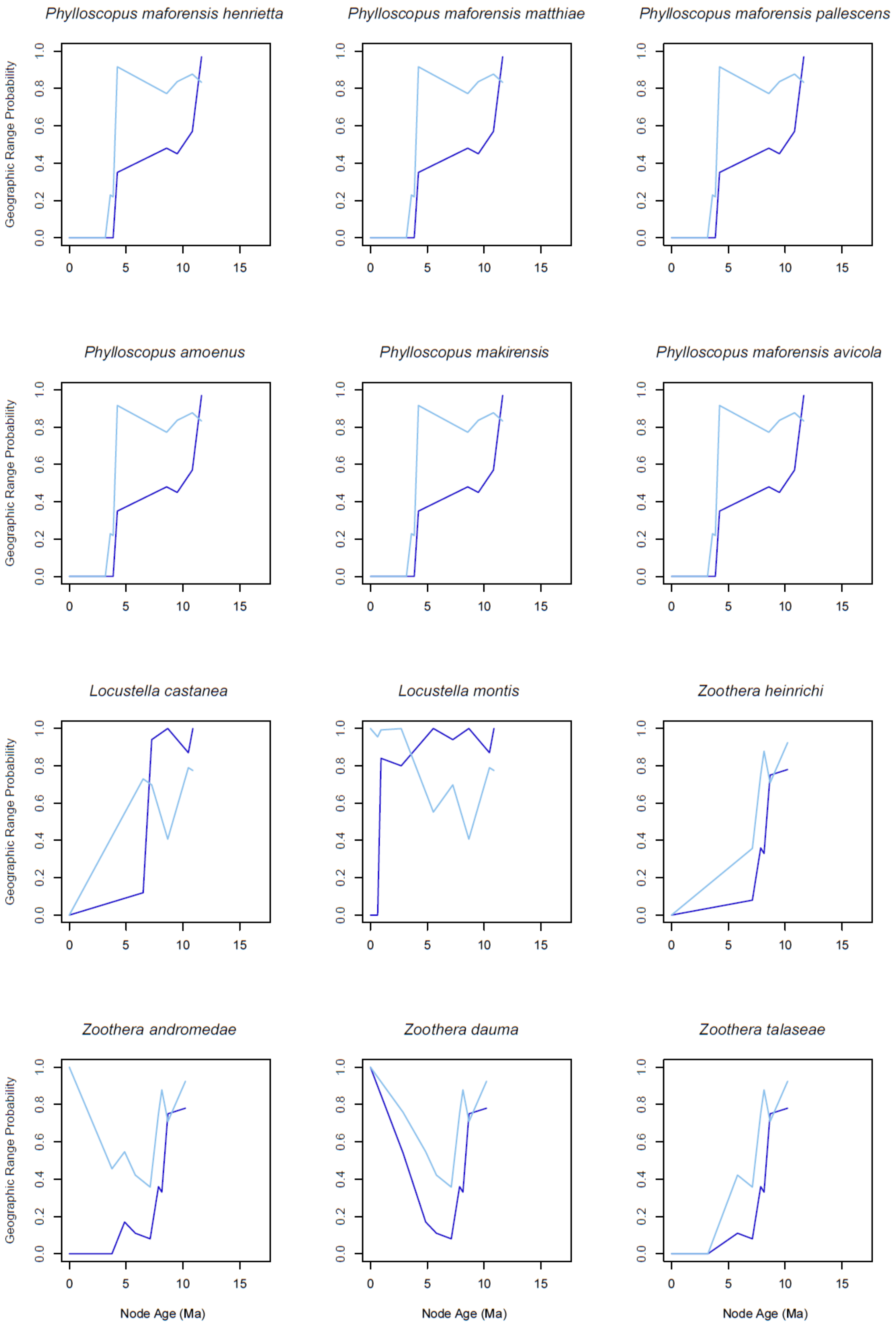

Supplementary Fig. 1b. Ancestral geographic range curves for Eurasian-origin species. Curves link composite region scores for nodes in the ancestral geographic range reconstructions. Dark blue lines connect posterior probabilities of Palearctic occurrence, light blue lines Indomalayan occurrence.

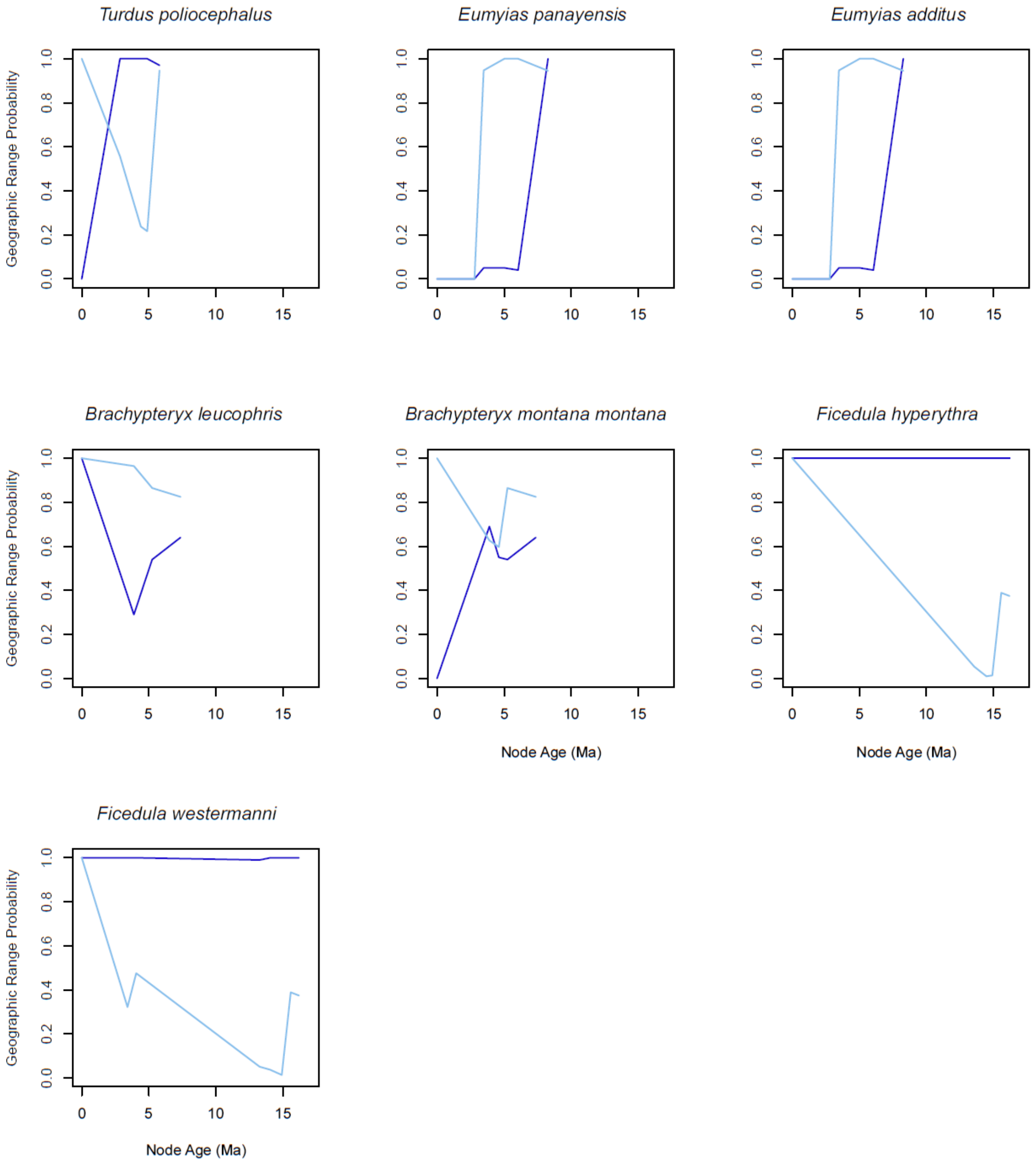

Supplementary Fig. 1c. Ancestral geographic range curves for Eurasian-origin species. Curves link composite region scores for nodes in the ancestral geographic range reconstructions. Dark blue lines connect posterior probabilities of Palearctic occurrence, light blue lines Indomalayan occurrence.

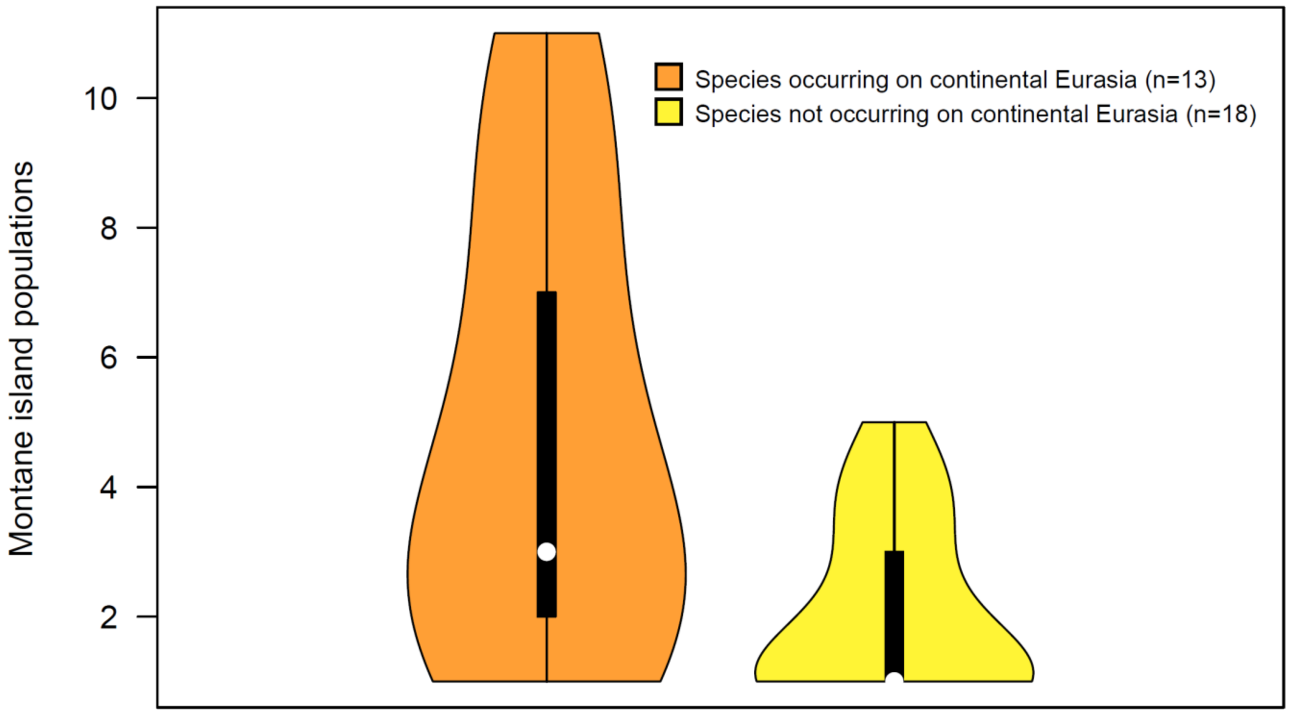

Supplementary Fig. 2. Number of montane island populations (MIPs) per Eurasian-origin species which respectively do, or do not, have ranges including continental Eurasia. The fact that species with continental occurrence have more MIPs may indicate that lineages' colonization capacities decline with longer residence in tropical archipelagos.

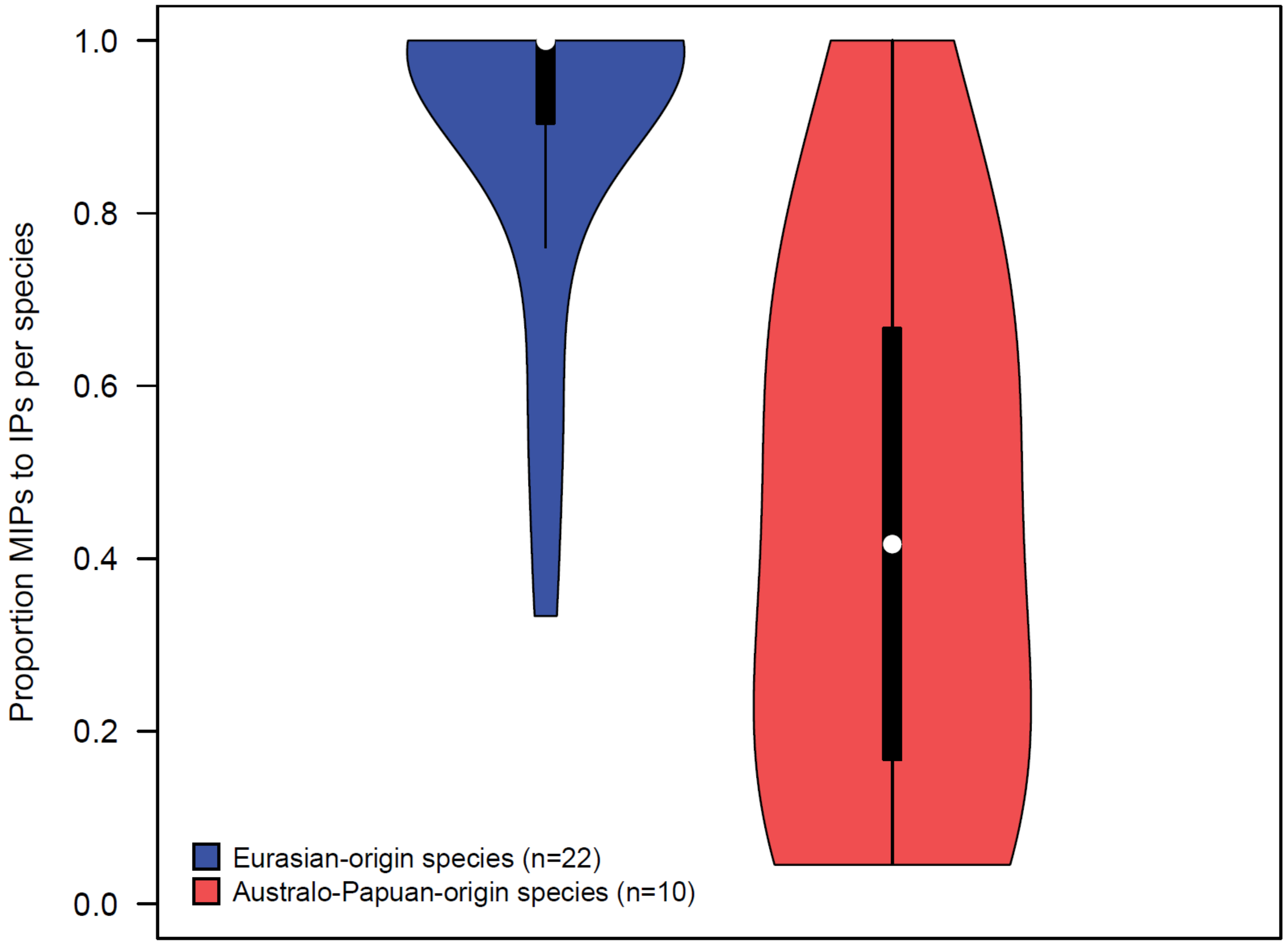

Supplementary Fig. 3. Proportion of montane island populations (MIPs) to total island populations (IPs) per species, for Eurasian-origin versus Australo-Papuan-origin species. Species with one MIP and no lowland island populations (LIPs) are excluded. Eurasian-origin species with MIPs are consistently montane across their archipelagic ranges, but Australo-Papuan-origin species are not.

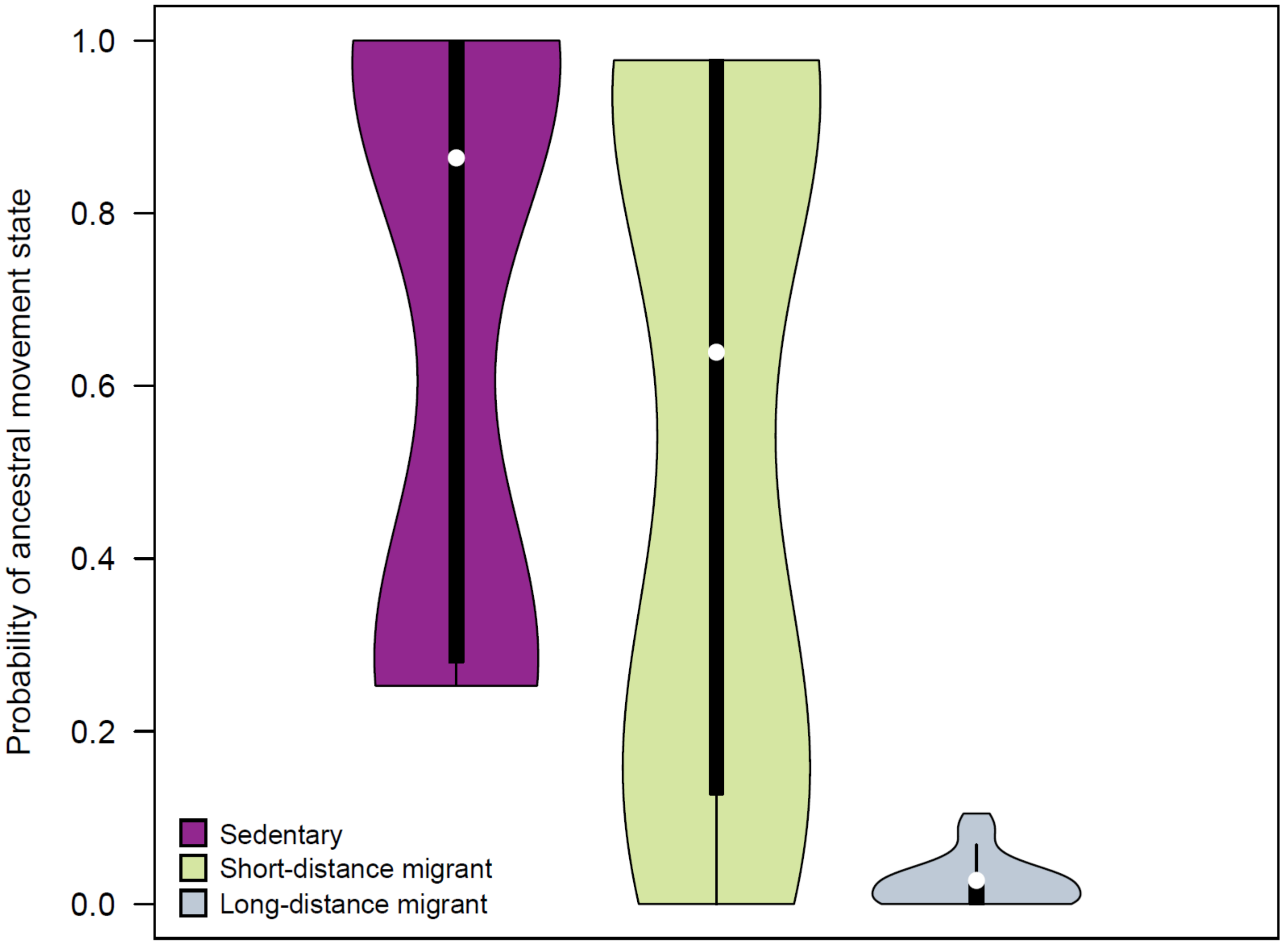

Supplementary Fig. 4. Probabilities of ancestral migratory behavior states for Eurasian-origin species with montane island populations (MIPs). Values are derived from the probabilities at the Ancestral Source Nodes of all species, as described in Methods: Migration. Note that migratory behavior classes are not mutually exclusive for species or ancestral nodes, as different populations within a single species can show different migratory behavior. Australo-Papuan-origin species are not shown because their continental ancestors were almost entirely sedentary.

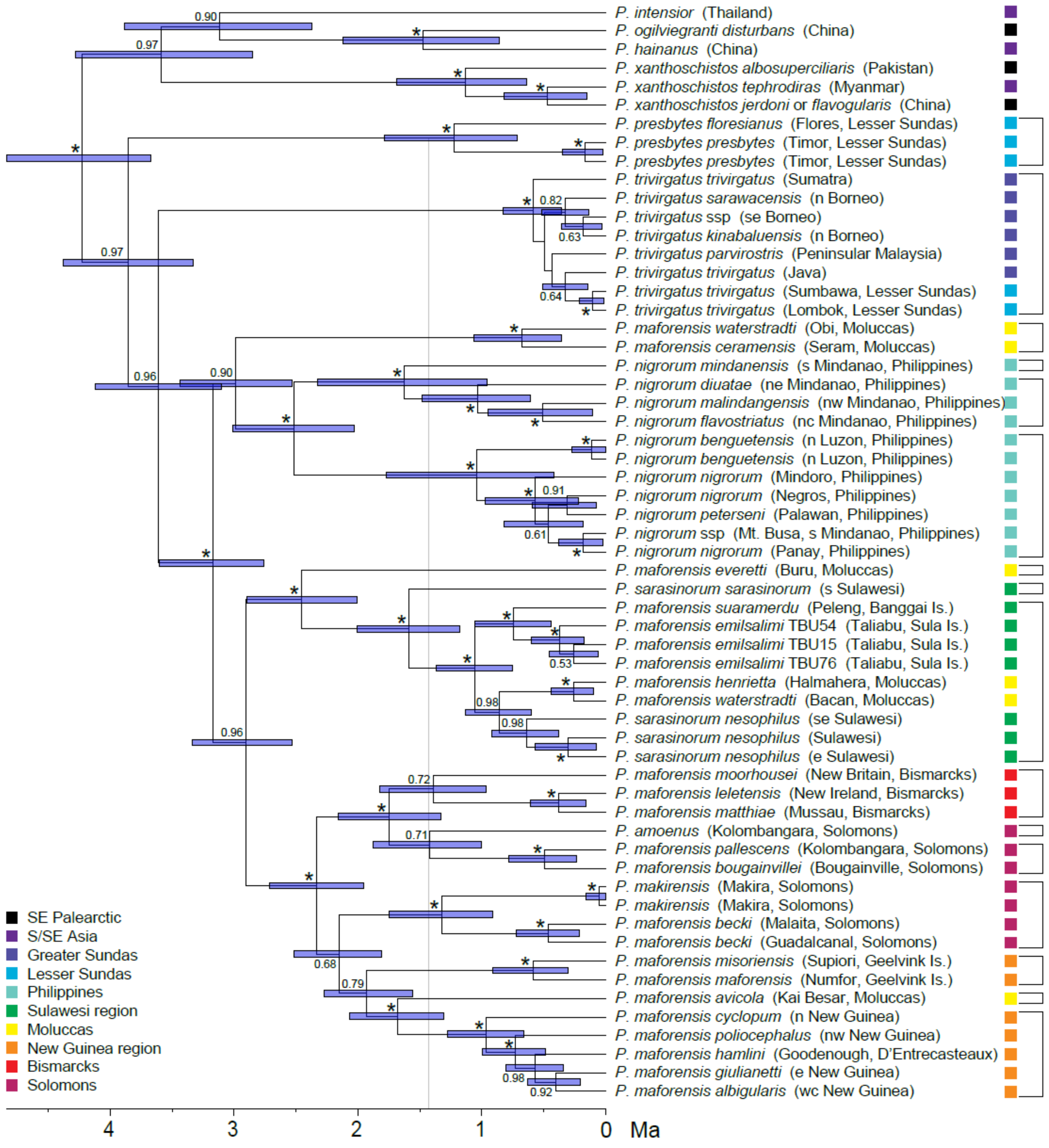

Supplementary Fig. 5. Indo-Pacific *Phylloscopus* leaf warblers: Time-calibrated Bayesian consensus tree from supermatrix analysis of three nuclear and two mitochondrial loci. Posterior probabilities ≥ 0.50 are indicated at the nodes; asterisks denote nodes with PP ≥ 0.99. Error bars indicate 95% highest posterior density (HPD) intervals. A four-species sister clade is shown, but other outgroups are not. Colored cells indicate areas of occurrence (breeding ranges). We used temporal banding to make a preliminary revision of species limits for use in this study. The threshold for species-level divergence was set at 1.42 Ma (indicated with a gray line) based on the divergence date estimate for the two sympatric species on Kolombangara, *P. amoenus* and *P. maforensis pallescens*. Species membership as defined for this study is indicated with brackets.

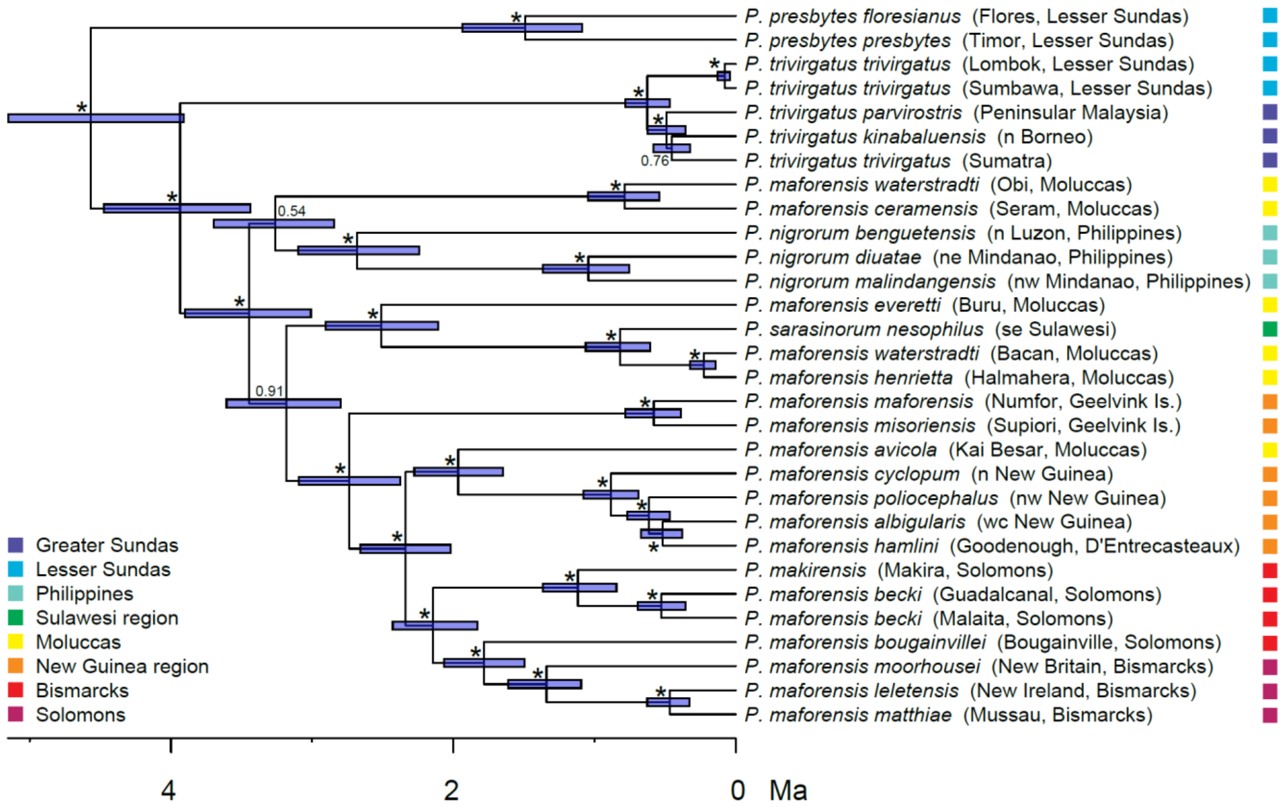

Supplementary Fig. 6. Indo-Pacific *Phylloscopus* leaf warblers: Time-calibrated Bayesian consensus tree from analysis of mitochondrial genomes. Posterior probabilities ≥ 0.50 are indicated at the nodes; asterisks denote nodes with PP ≥ 0.99. Error bars indicate 95% highest posterior density (HPD) intervals. The single outgroup taxon is not shown. Colored cells indicate areas of occurrence (breeding ranges).

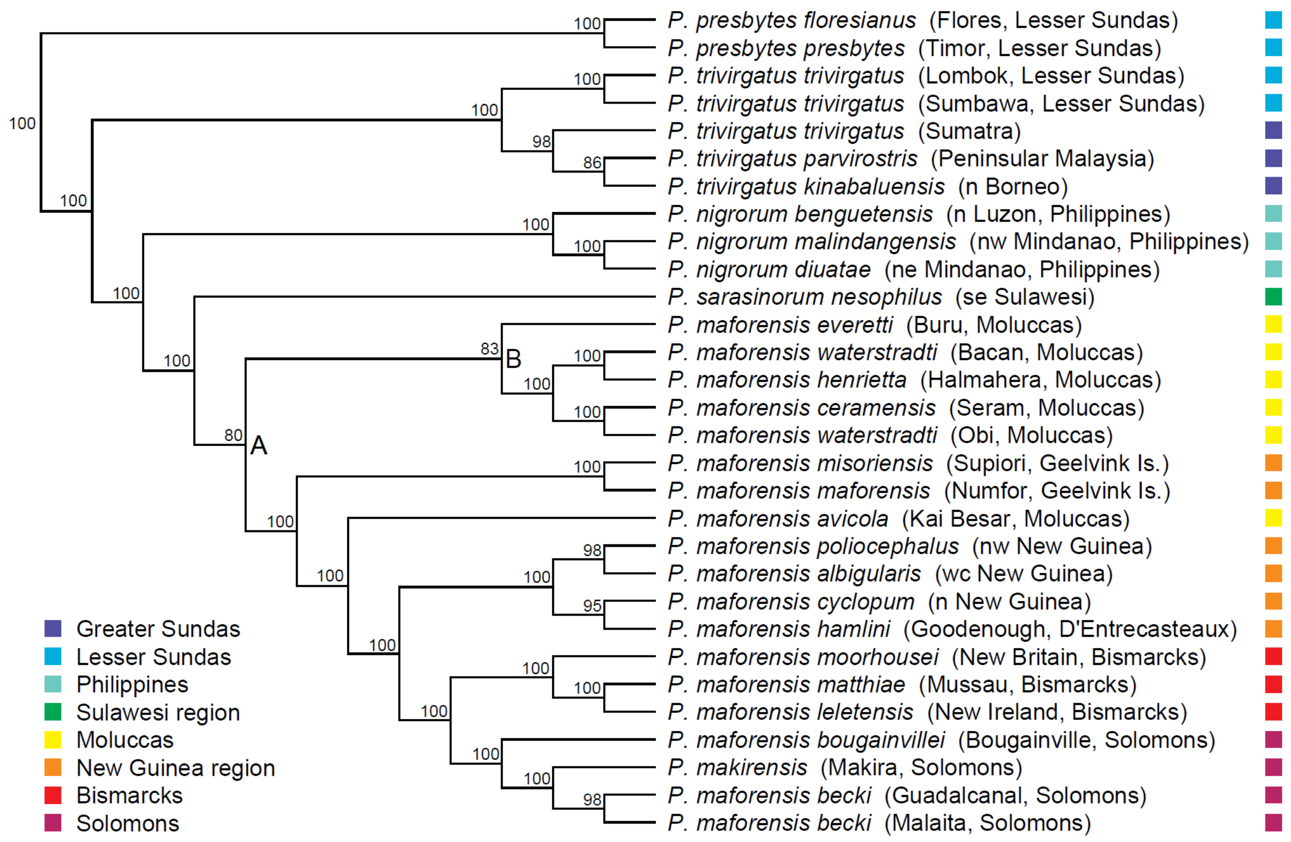

Supplementary Fig. 7. Indo-Pacific *Phylloscopus* leaf warblers: Concatenated genomic maximum likelihood tree. ML bootstrap supports ≥ 50 are indicated at the nodes. Nodes labelled with letters are discussed in the text. Outgroup taxa are not shown. Colored cells indicate areas of occurrence (breeding ranges).

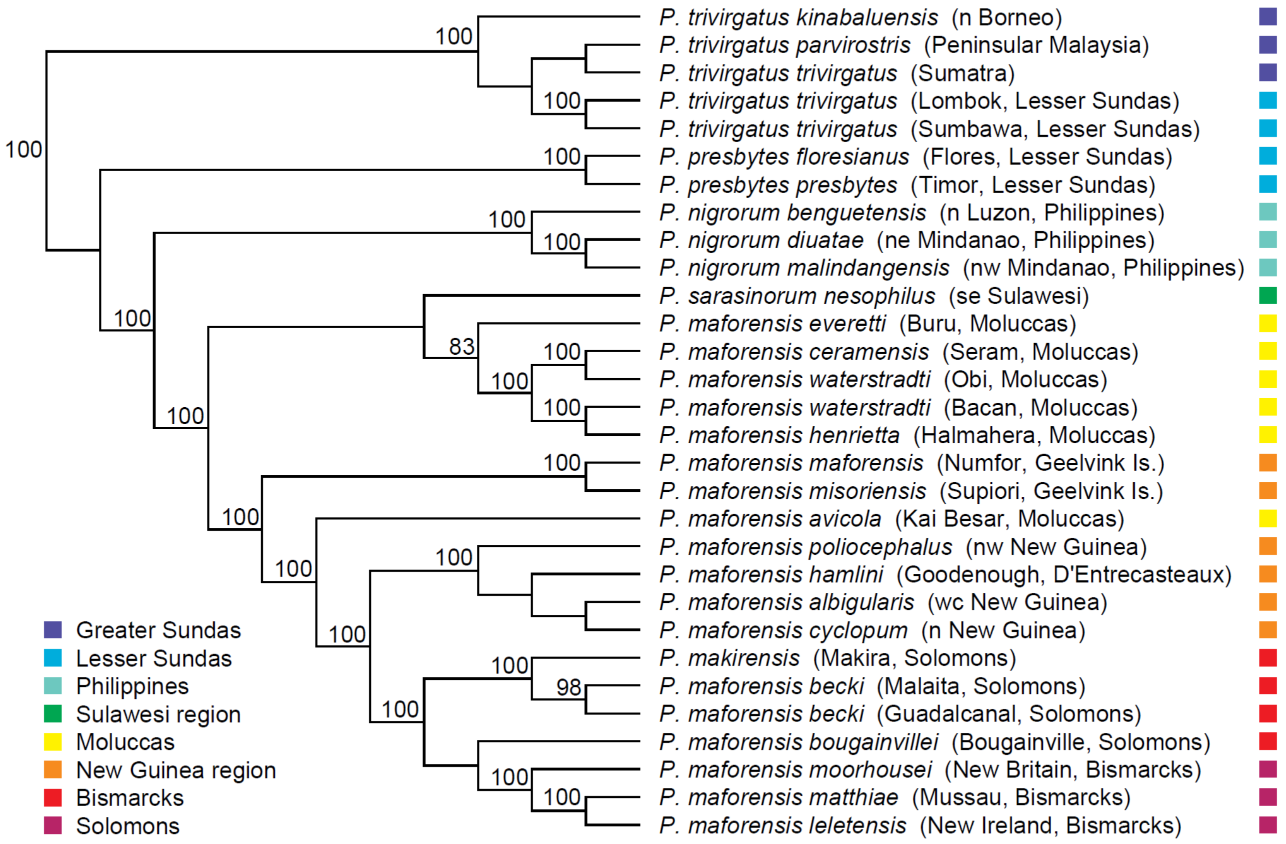

Supplementary Fig. 8. Indo-Pacific *Phylloscopus* leaf warblers: Genomic species tree with maximum likelihood bootstrap values. ML bootstrap supports ≥ 50 are indicated at the nodes. Outgroup taxa are not shown. Colored cells indicate areas of occurrence (breeding ranges).

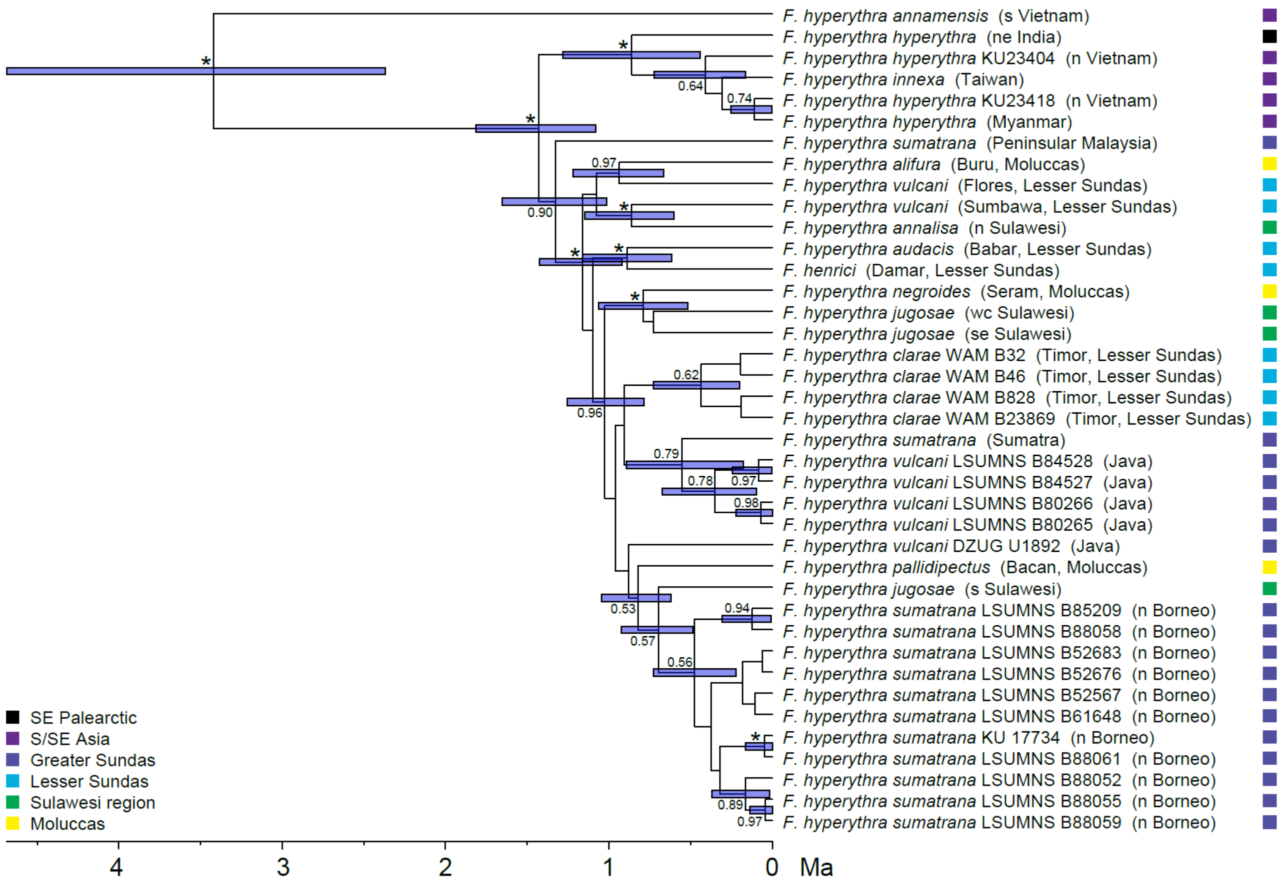

Supplementary Fig. 9. Snowy-browed Flycatcher *Ficedula hyperythra*: time-calibrated Bayesian consensus tree from supermatrix analysis of three nuclear and three mitochondrial loci. Posterior probabilities ≥ 0.50 are indicated at the nodes; asterisks denote nodes with PP ≥ 0.99. Error bars indicate 95% highest posterior density (HPD) intervals. Outgroup taxa are not shown. Colored cells indicate areas of occurrence (breeding ranges).

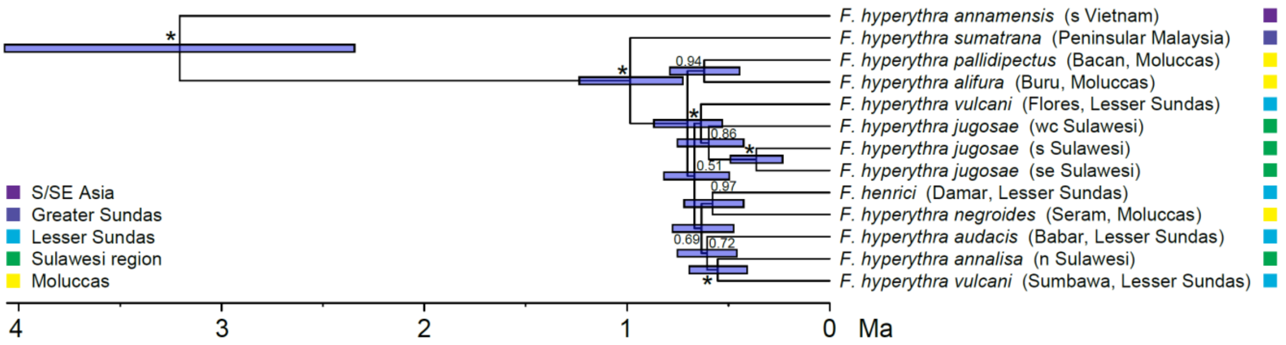

Supplementary Fig. 10. Snowy-browed Flycatcher *Ficedula hyperythra*: Time-calibrated Bayesian consensus tree from analysis of mitochondrial genomes. Posterior probabilities ≥ 0.50 are indicated at the nodes; asterisks denote nodes with PP ≥ 0.99. Error bars indicate 95% highest posterior density (HPD) intervals. Outgroup taxa are not shown. Colored cells indicate areas of occurrence (breeding ranges).

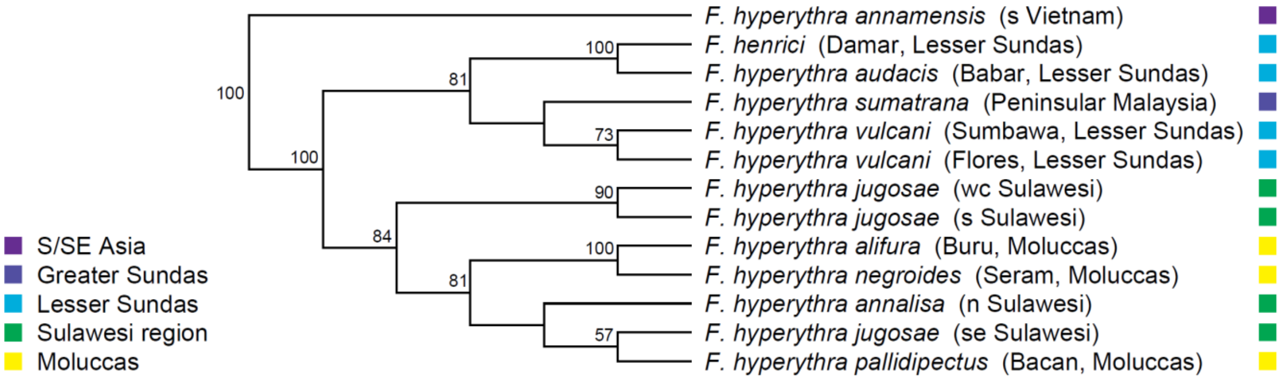

Supplementary Fig. 11. Snowy-browed Flycatcher *Ficedula hyperythra*: Concatenated genomic maximum likelihood tree. ML bootstrap supports ≥ 50 are indicated at the nodes. Outgroup taxa are not shown. Colored cells indicate areas of occurrence (breeding ranges).

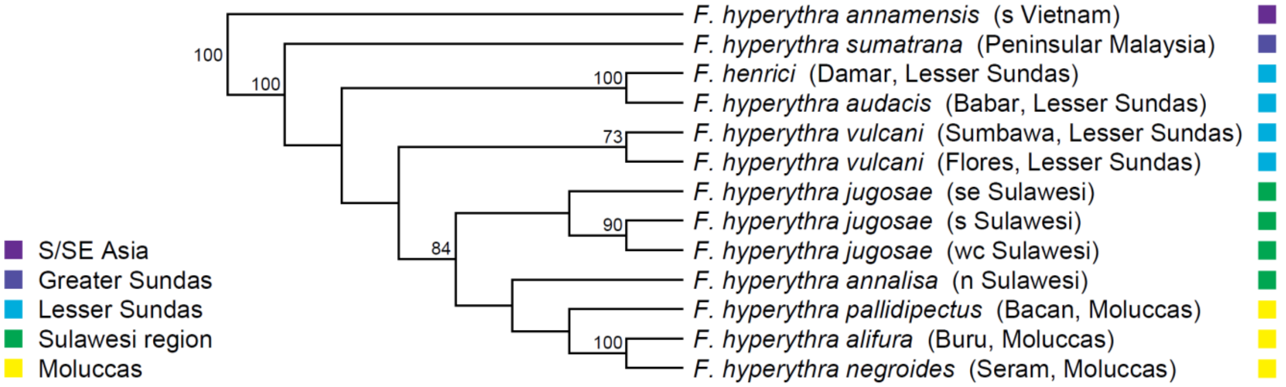

Supplementary Fig. 12. Snowy-browed Flycatcher *Ficedula hyperythra*: Genomic species tree with maximum likelihood bootstrap values. ML bootstrap supports ≥ 50 are indicated at the nodes. Outgroup taxa are not shown. Colored cells indicate areas of occurrence (breeding ranges).

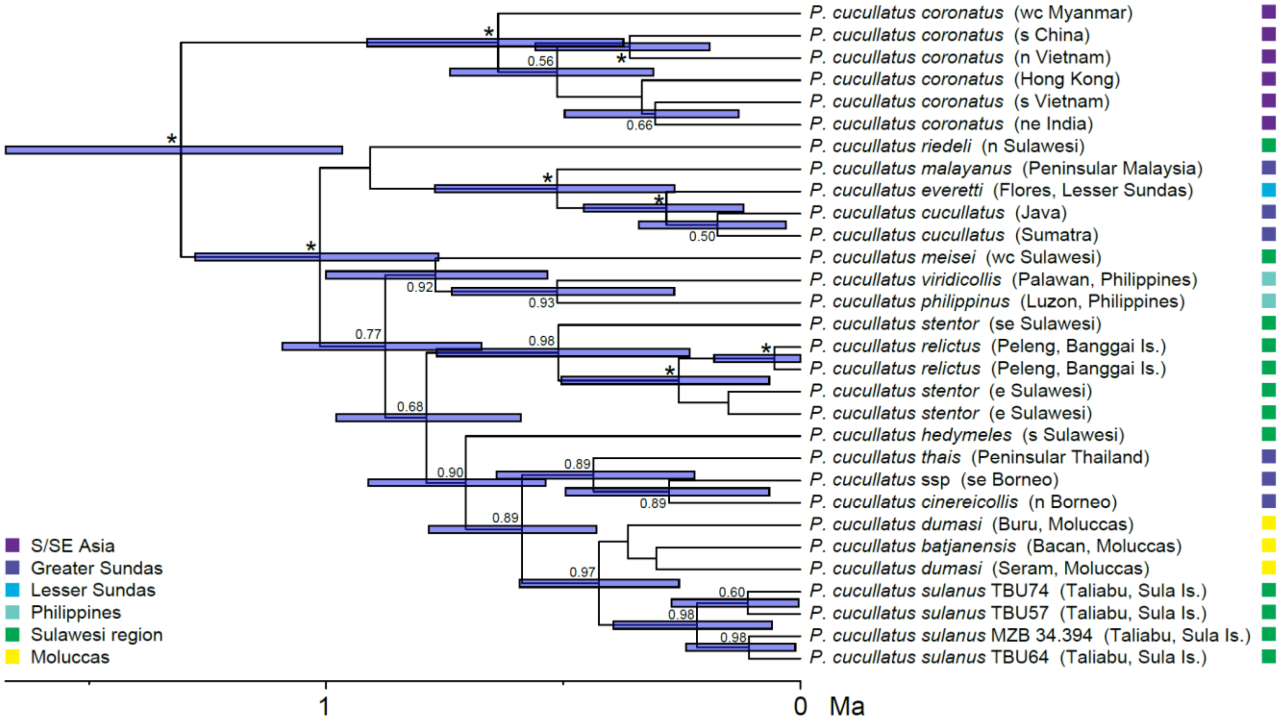

Supplementary Fig. 13. Mountain Tailorbird *Phyllergates cucullatus*: time-calibrated Bayesian consensus tree from supermatrix analysis of five nuclear and two mitochondrial loci. Posterior probabilities ≥ 0.50 are indicated at the nodes; asterisks denote nodes with PP ≥ 0.99. Error bars indicate 95% highest posterior density (HPD) intervals. Outgroup taxa are not shown. Colored cells indicate areas of occurrence (breeding ranges).

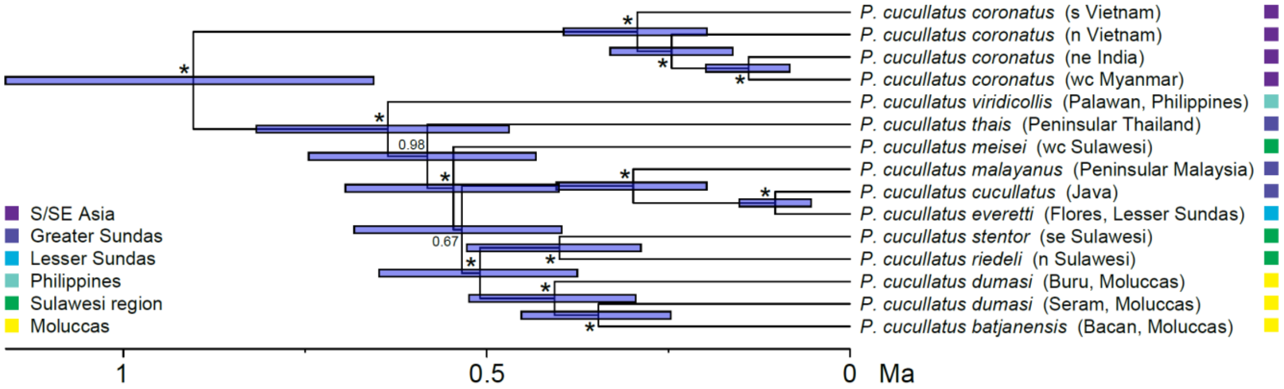

Supplementary Fig. 14. Mountain Tailorbird *Phyllergates cucullatus*: Time-calibrated Bayesian consensus tree from analysis of mitochondrial genomes. Posterior probabilities ≥ 0.50 are indicated at the nodes; asterisks denote nodes with PP ≥ 0.99. Error bars indicate 95% highest posterior density (HPD) intervals. The single outgroup taxon is not shown. Colored cells indicate areas of occurrence (breeding ranges).

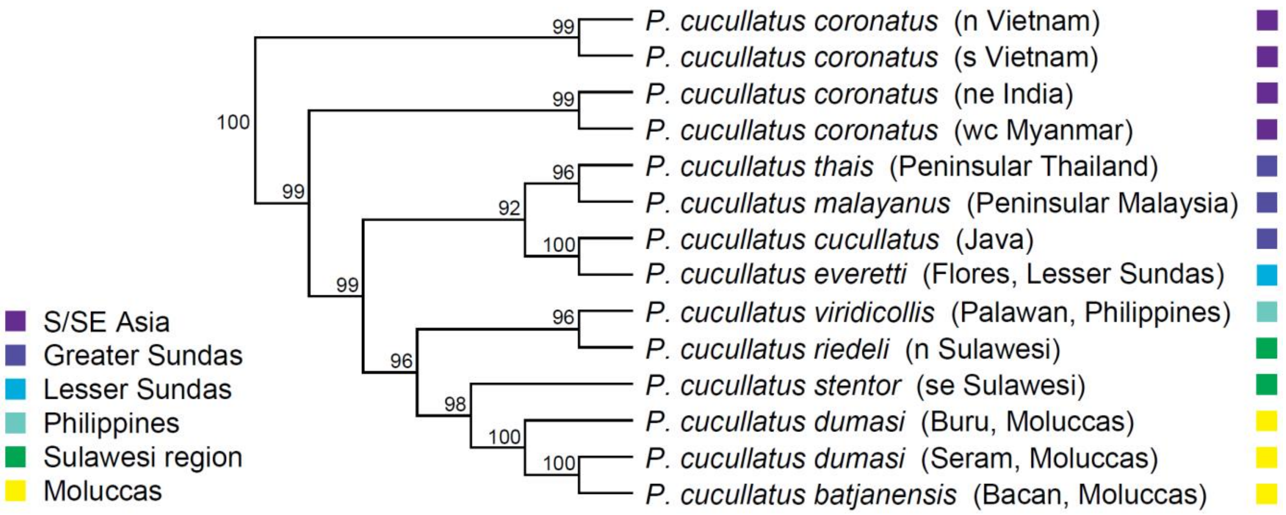

Supplementary Fig. 15. Mountain Tailorbird *Phyllergates cucullatus*: Concatenated genomic maximum likelihood tree. ML bootstrap supports ≥ 50 are indicated at the nodes. Outgroup taxa are not shown. Colored cells indicate areas of occurrence (breeding ranges).

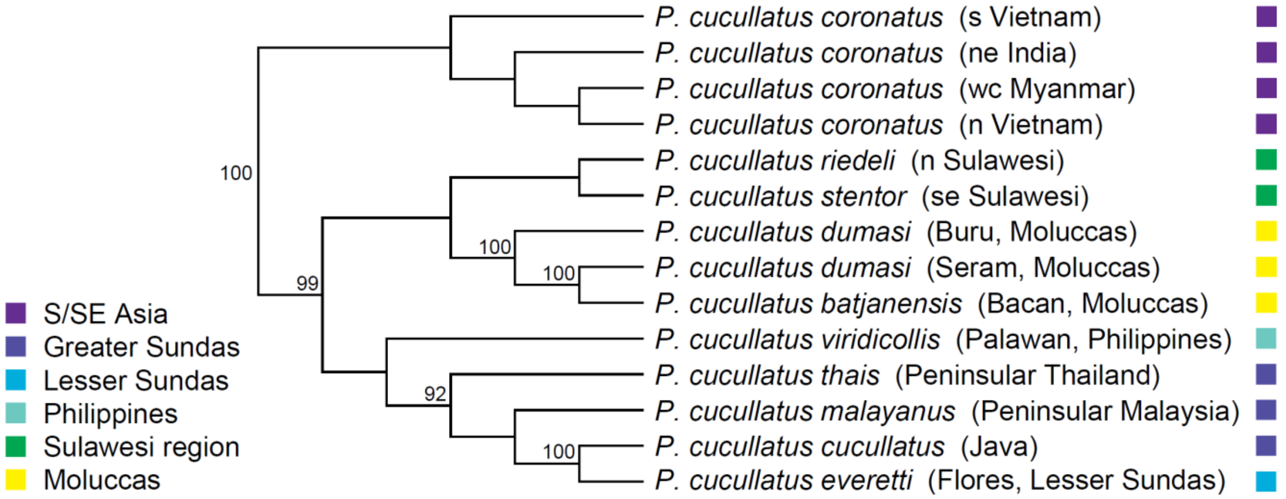

Supplementary Fig. 16. Mountain Tailorbird *Phyllergates cucullatus*: Genomic species tree with maximum likelihood bootstrap values. ML bootstrap supports ≥ 50 are indicated at the nodes. Outgroup taxa are not shown. Colored cells indicate areas of occurrence (breeding ranges).

SUPPLEMENTARY TABLES

Supplementary Table 1. Taxonomic diversity of species with montane island population (MIPs).

	Genera	Families
Groups identified	47	22
Groups placed in our trees	37	17
representation by Eurasian-origin species	11	7
representation by Australo-Papuan-origin species	14	5
representation by other species	14	11

Supplementary Table 2. Models used for ancestral state reconstructions. AIC values are shown for DEC and DECj models for the analyses of geographic range, elevational range, and migration across all clades. The DECj model was used if its AIC value was > 2 under the AIC value of the DEC model. A "-" in a cell indicates that no analysis was performed because all species had the same character state.

Clade	Geographic Range		Elevational Range		Migration	
	DEC	DECj	DEC	DECj	DEC	DECj
Meliphagidae	475.50	382.10	311.30	258.30	203.90	205.90
Campephagidae	412.90	713.10	142.90	120.80	102.70	104.70
Pachycephalidae	258.20	229.50	127.63	107.78	35.85	37.85
Rhipiduridae A (*Rhipidura*)	87.83	77.48	36.51	34.91	28.34	30.34
Rhipiduridae B (Lamproliidae)	15.12	11.72	11.17	9.66	-	-
Corvidae	141.90	136.00	16.97	15.82	74.83	75.70
Petroicidae A (*Microeca*)	21.08	21.06	21.88	17.37	-	-
Petroicidae B (*Petroica*)	37.25	34.80	28.88	26.88	30.38	32.38
Stenostiridae	37.59	39.56	21.66	23.66	15.30	17.30
Pnoepygidae	14.84	16.77	-	-	7.64	9.64
Cettiidae	190.30	175.30	78.28	67.01	84.43	86.43
Phylloscopidae	145.61	151.80	56.82	59.08	96.53	94.53
Locustellidae	266.60	650.80	124.90	99.71	156.00	153.00
Sturnidae	146.90	133.10	40.49	35.28	26.34	28.34
Turdidae A (*Geokichla*)	35.62	36.99	34.81	25.84	16.88	18.88
Turdidae B (*Zoothera*)	116.20	108.70	34.30	36.30	62.55	121.40
Turdidae C (*Turdus*)	55.14	57.14	60.28	55.30	103.90	105.90
Muscicapidae A (*Eumyias*)	32.31	31.12	16.28	17.49	21.56	23.56
Muscicapidae B (*Brachypteryx*)	46.11	60.50	-	-	15.66	20.27
Muscicapidae C (*Ficedula*)	103.20	101.80	87.96	67.18	79.58	79.75
Dicaeidae	102.26	88.08	31.48	27.05	23.39	25.39
Motacillidae	41.03	34.93	26.04	26.95	44.80	43.19
Fringillidae	54.73	54.26	70.78	61.75	122.30	124.30

Supplementary Table 3. Establishment of multiple montane island populations (MIPs) by Eurasian-origin vs. Australo-Papuan-origin species.

	All archipelagos		Wallacea		Bismarcks/Solomons	
No. MIPs	mean	median	mean	median	mean	median
Eurasian-origin species	3.23	2	3.29	2	2.63	2.5
Australo-Papuan-origin species	1.32	1	1.55	1	1.14	1
Proportion of spp with >1 MIP						
Eurasian-origin species	0.65		0.67		0.63	
Australo-Papuan-origin species	0.2		0.36		0.07	

Supplementary Table 4. Statistical testing of differences in Supplementary Table 3: multiple MIP establishment by Eurasian-origin vs. Australo-Papuan-origin species. Results of Mann-Whitney U-tests (two-sided) and chi-squared tests are given. Tests were repeated across different regions: all archipelagos (Wal/Bis/Sol); Wallacea (Wal); and the Bismarcks and Solomons (Bis/Sol).

Measure	Region	Test	W	Chi-sq	df	p
No. MIPs	Wal/Bis/Sol	M-W U-Test	581.5	NA	NA	0.0004472
	Wal		188			0.0385
	Bis/Sol		87.5			0.00692
Frequency of spp with >1 MIPs	Wal/Bis/Sol	Chi-sq Test	NA	9.3692	1	0.002207
	Wal			1.7262	1	0.1889
	Bis/Sol			5.322	1	0.02106

Supplementary Table 5. Results for comparisons of empirical vs phylogenetic null datasets. Abbreviations: MIPs = montane island populations; IPs = total island populations; EUR = Eurasian-origin species; AP = Australo-Papuan-origin species; SDM = short-distance migration; SED = sedentary; CON = continental occurrence; ISL = no continental occurrence. F statistics from one-way ANOVA tests of empirical data are given, as well as the 5% upper quantiles of the distributions of the simulated F statistics.

Analysis	Empirical Value of F	Null upper 5%	p
Montane vs. lowland ancestry: EUR vs. AP	107.1	36.91108	0.001
No. MIPs (total): EUR vs. AP	11	14.37757	0.071
No. MIPs (Wallacea): EUR vs. AP	4.224	8.359259	0.158
No. MIPs (Bismarcks/Solomons): EUR vs. AP	8.741	11.51451	0.076
MIPs/IPs: EUR vs. AP	25.96	14.82485	0.012
Migration ancestry: EUR vs. AP	49.01	40.22154	0.029
No. MIPs (total): EUR SDM vs. SED	7.866	5.979078	0.024
No. MIPs (total): EUR CON vs. ISL	9.761	7.742936	0.025

Supplementary Table 6. Taxon sampling of montane supercolonizers.

Clade	No. taxa	No. taxa our sampling	No. individuals our sampling	No. taxa Genbank	No. individuals Genbank	No. taxa supermatrix	No. individuals supermatrix
Indo-Pacific *Phylloscopus*	38	26 (68%)	30	16	23	37 (97%)	53
Phylloscopus amoenus	1	0 (0%)	0	1	1	1 (100%)	1
Phylloscopus maforensis	20	16 (80%)	18	4	6	20 (100%)	24
Phylloscopus makirensis	1	1 (100%)	1	1	1	1 (100%)	2
Phylloscopus nigrorum	7	3 (43%)	3	5	8	7 (100%)	11
Phylloscopus presbytes	2	2 (100%)	2	1	1	2 (100%)	3
Phylloscopus rotiensis	1	0 (0%)	0	0	0	0 (0%)	0
Phylloscopus sarasinorum	2	1 (50%)	1	2	3	2 (100%)	4
Phylloscopus trivirgatus	4	3 (75%)	5	2	3	4 (100%)	8
Ficedula hyperythra	14	9 (64%)	12	6	26	13 (93%)	38
Phyllergates cucullatus	16	11 (69%)	15	8	15	16 (100%)	30

Supplementary Table 7. Reference sequences for gene extraction.

Study taxon	Reference sequences
Phylloscopus spp	mitochondrial genome: *Phylloscopus examinandus* LR026996
	Myo: *Phylloscopus claudiae* MH079093
	GAPDH: *Phylloscopus claudiae* MH042994
	ODC: *Phylloscopus claudiae* MH037419
Ficedula spp	mitochondrial genome: *Ficedula zanthopygia* JN018411
	Myo: *Ficedula tricolor* KJ931278
	ODC: *Ficedula tricolor* KJ931305
	PEPCK: *Ficedula mugimaki* KJ931247
Phyllergates cucullatus	mitochondrial genome: *Aegithalos glaucogularis* KF951090
	Myo: *Tickellia hodgsoni* DQ008565
	GAPDH: *Tickellia hodgsoni* HQ121538
	ODC: *Tickellia hodgsoni* EU680774
	TGFb2: *Phyllergates heterolaemus* JX006177
	MUSK: *Phyllergates heterolaemus* JX006218
Horornis parens	mitochondrial genome: *Horonis fortipes* MK051002
Poodytes albolimbatus	mitochondrial genome: *Poodytes punctatus* NC_029138
Aplonis mystacea	mitochondrial genome: *Acridotheres tristis* HQ915864
Dicaeum pectorale	mitochondrial genome: *Aethopyga gouldiae* NC_027241

SUPPLEMENTARY REFERENCES

1. Price, T. D. The roles of time and ecology in the continental radiation of the Old World leaf warblers (*Phylloscopus* and *Seicercus*). *Philos. Trans. R. Soc. Lond. B Biol. Sci.* **365,** 1749-1762 (2010).

2. Alström, P. et al. Complete species-level phylogeny of the leaf warbler (Aves: Phylloscopidae) radiation. *Mol. Phylogenet. Evol.* **126,** 141-152 (2018).

3. Jones, A. W. & Kennedy, R. S. Evolution in a tropical archipelago: comparative phylogeography of Philippine fauna and flora reveals complex patterns of colonization and diversification. *Biol. J. Linn. Soc. Lond.* **95,** 620-639 (2008).

4. Avise, J. C. & Johns, G. C. Proposal for a standardized temporal scheme of biological classification for extant species. *Proc. Natl. Acad. Sci. U.S.A.* **96,** 7358-7363 (1999).

5. Eames, J. C., Steinheimer, F. D. & Bansok, R. A collection of birds from the Cardamom Mountains, Cambodia, including a new subspecies of *Arborophila cambodiana*. *Forktail* **18,** 67-86 (2002).

6. Moyle, R. G., Hosner, P. A., Jones, A. W. & Outlaw, D. C. Phylogeny and biogeography of *Ficedula* flycatchers (Aves: Muscicapidae): novel results from fresh source material. *Mol. Phylogenet. Evol.* **82,** 87-94 (2015).

7. Hooper, D. M., Olsson, U. & Alström, P. The Rusty-tailed Flycatcher (*Muscicapa ruficauda*; Aves: Muscicapidae) is a member of the genus *Ficedula*. *Mol. Phylogenet. Evol.* **102,** 56-61 (2016).

8. Trainor, C. R. & Verbelen, P. New distributional records from forgotten Banda Sea islands: the birds of Babar, Romang, Sermata, Leti and Kisar, Maluku, Indonesia. *Bull. Br. Ornithol. Club* **133,** 272-317 (2013).

9. Trainor, C. R. Birds of Damar Island, Banda Sea, Indonesia. *Bull. Br. Ornithol. Club* **127,** 300-321 (2007).
