# Supplementary material for: The formation of the Indo-Pacific montane avifauna
Source: Nat Commun. 2023 Dec 11;14:8215. doi: 10.1038/s41467-023-43964-y (PMC10713610; doi:10.1038/s41467-023-43964-y)
Supplement: Supplementary file 3 — Description of Additional Supplementary Files [file 41467_2023_43964_MOESM3_ESM.pdf]

DESCRIPTION OF ADDITIONAL SUPPLEMENTARY FILES

Supplementary Data 1. Species distributions, region assignments, co-occurrence patterns, migratory behavior, and ancestral elevational distribution and migratory behavior.

Supplementary Data 2. Species-level phylogenetic trees.

Supplementary Data 3. Tree files.

Supplementary Data 4. Ancestral state reconstructions: geographic range, elevational range, and migratory behavior.

Supplementary Data 5. Species-level phylogenetic analyses: gene sampling, settings, etc.

Supplementary Data 6. Specimen and sequence data accession information.

Supplementary Data 7. List of primers.
