# Supplementary material for: The formation of the Indo-Pacific montane avifauna
Source: Nat Commun. 2023 Dec 11;14:8215. doi: 10.1038/s41467-023-43964-y (PMC10713610; doi:10.1038/s41467-023-43964-y)
Supplement: Supplementary file 5 — Supplementary Data 2 [file 41467_2023_43964_MOESM5_ESM.pdf]

Supplementary Material for

The formation of the Indo-Pacific montane avifauna

Andrew Hart Reeve, Jonathan David Kennedy, Jose Martin Pujolar, Bent Petersen, Mozes P. K. Blom, Per Alström, Tri Haryoko, Per G. P. Ericson, Martin Irestedt, Johan A. A. Nylander, and Knud Andreas Jønsson

Supplementary Data 2. Species-level trees. Black circles at nodes indicate posterior probabilities of 0.99-1.00; gray circles indicate PP 0.95-0.98; and white circles indicate PP 0.90-0.94. Nodes without circles have PP < 0.90.

Contents:

2. Pachycephalidae - p. 2
3. Petroicidae A (*Microeca*) - p. 3
4. Petroicidae B (*Petroica*) - p. 4
5. Stenostiridae - p. 5
6. Pnoepygidae - p. 6
7. Cettiidae - p. 7
8. Locustellidae - p. 8
9. Sturnidae - p. 9
10. Turdidae A (*Geokichla*) - p. 10
11. Turdidae B (*Zoothera*) - p. 11
12. Turdidae C (*Turdus*) - p. 12
13. Muscicapidae A (*Eumyias*) - p. 13
14. Muscicapidae B (*Brachypteryx*) - p. 14
15. Muscicapidae C (*Ficedula*) - p. 15
16. Dicaeidae - p. 16
17. Fringillidae - p. 17

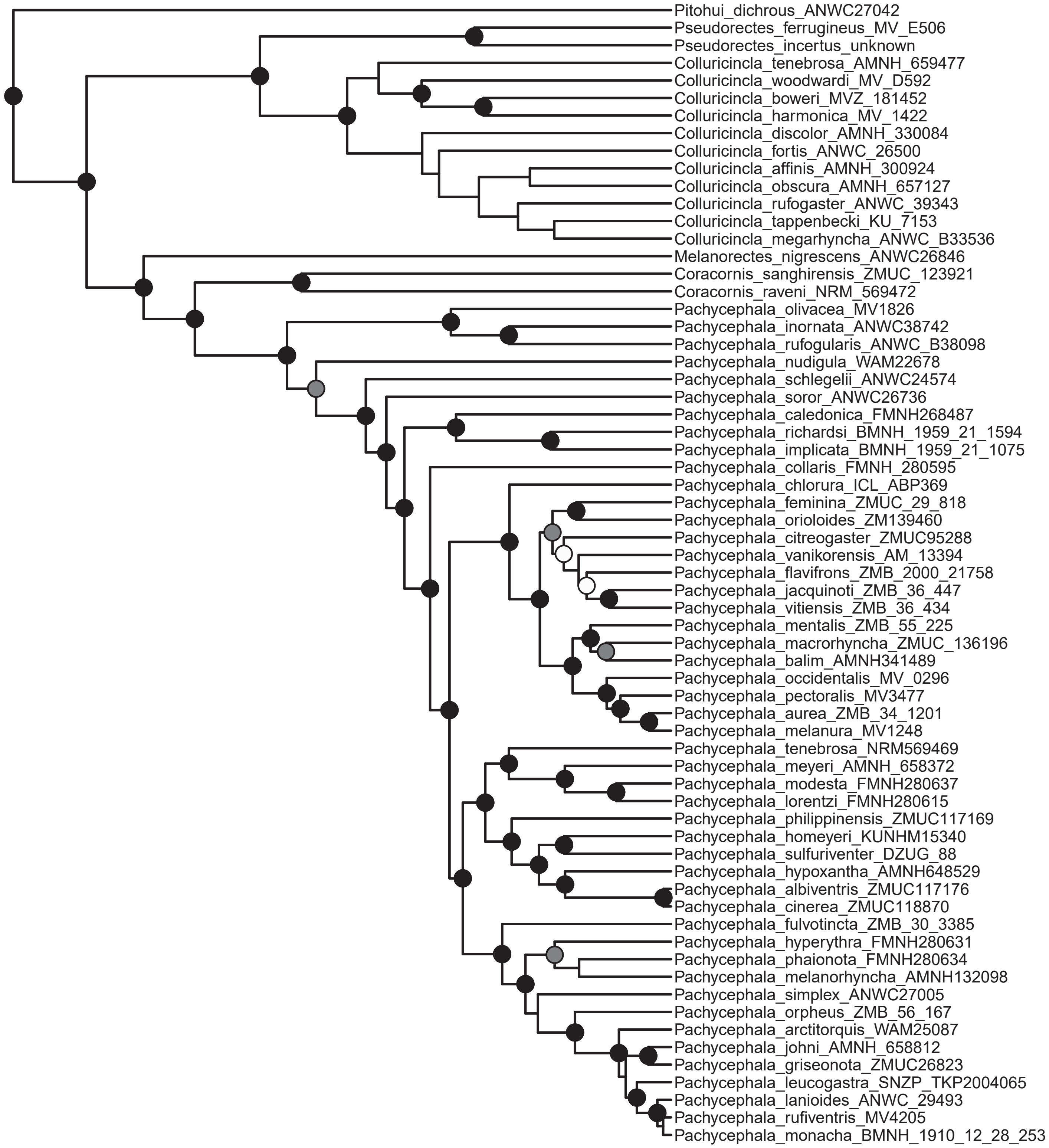

Pitohui_dichrous_ANWC27042
Pseudorectes_ferrugineus_MV_E506
Pseudorectes_incertus_unknown
Colluricincla_tenebrosa_AMNH_659477
Colluricincla_woodwardi_MV_D592
Colluricincla_boweri_MVZ_181452
Colluricincla_harmonica_MV_1422
Colluricincla_discolor_AMNH_330084
Colluricincla_fortis_ANWC_26500
Colluricincla_affinis_AMNH_300924
Colluricincla_obscura_AMNH_657127
Colluricincla_rufogaster_ANWC_39343
Colluricincla_tappenbecki_KU_7153
Colluricincla_megarhyncha_ANWC_B33536
Melanorectes_nigrescens_ANWC26846
Coracornis_sanghirensis_ZMUC_123921
Coracornis_raveni_NRM_569472
Pachycephala_olivacea_MV1826
Pachycephala_inornata_ANWC38742
Pachycephala_rufogularis_ANWC_B38098
Pachycephala_nudigula_WAM22678
Pachycephala_schlegelii_ANWC24574
Pachycephala_soror_ANWC26736
Pachycephala_caledonica_FMNH268487
Pachycephala_richardsi_BMNH_1959_21_1594
Pachycephala_implicata_BMNH_1959_21_1075
Pachycephala_collaris_FMNH_280595
Pachycephala_chlorura_ICL_ABP369
Pachycephala_feminina_ZMUC_29_818
Pachycephala_orioloides_ZM139460
Pachycephala_citreogaster_ZMUC95288
Pachycephala_vanikorensis_AM_13394
Pachycephala_flavifrons_ZMB_2000_21758
Pachycephala_jacquinoti_ZMB_36_447
Pachycephala_vitiensis_ZMB_36_434
Pachycephala_mentalis_ZMB_55_225
Pachycephala_macrorhyncha_ZMUC_136196
Pachycephala_balim_AMNH341489
Pachycephala_occidentalis_MV_0296
Pachycephala_pectoralis_MV3477
Pachycephala_aurea_ZMB_34_1201
Pachycephala_melanura_MV1248
Pachycephala_tenebrosa_NRM569469
Pachycephala_meyeri_AMNH_658372
Pachycephala_modesta_FMNH280637
Pachycephala_lorentzi_FMNH280615
Pachycephala_philippinensis_ZMUC117169
Pachycephala_homeyeri_KUNHM15340
Pachycephala_sulfuriventer_DZUG_88
Pachycephala_hypoxantha_AMNH648529
Pachycephala_albiventris_ZMUC117176
Pachycephala_cinerea_ZMUC118870
Pachycephala_fulvotincta_ZMB_30_3385
Pachycephala_hyperythra_FMNH280631
Pachycephala_phaionota_FMNH280634
Pachycephala_melanorhyncha_AMNH132098
Pachycephala_simplex_ANWC27005
Pachycephala_orpheus_ZMB_56_167
Pachycephala_arctitorquis_WAM25087
Pachycephala_johni_AMNH_658812
Pachycephala_griseonota_ZMUC26823
Pachycephala_leucogastra_SNZP_TKP2004065
Pachycephala_lanioides_ANWC_29493
Pachycephala_rufiventris_MV4205
Pachycephala_monacha_BMNH_1910_12_28_253

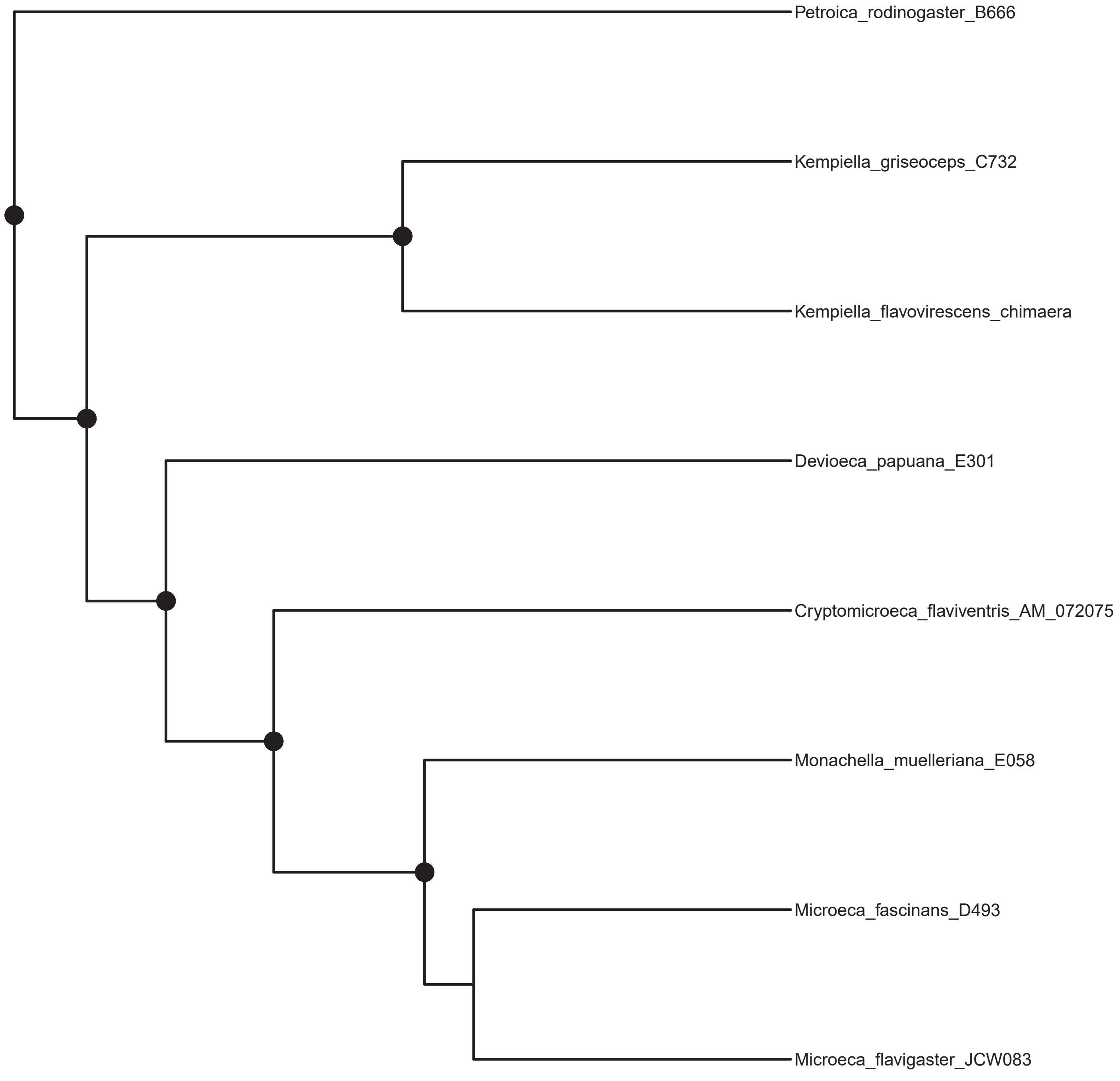
Petroica_rodinogaster_B666
Kempiella_griseoceps_C732
Kempiella_flavovirescens_chimaera
Devioeca_papuana_E301
Cryptomicroeca_flaviventris_AM_072075
Monachella_muelleriana_E058
Microeca_fascinans_D493
Microeca_flavigaster_JCW083

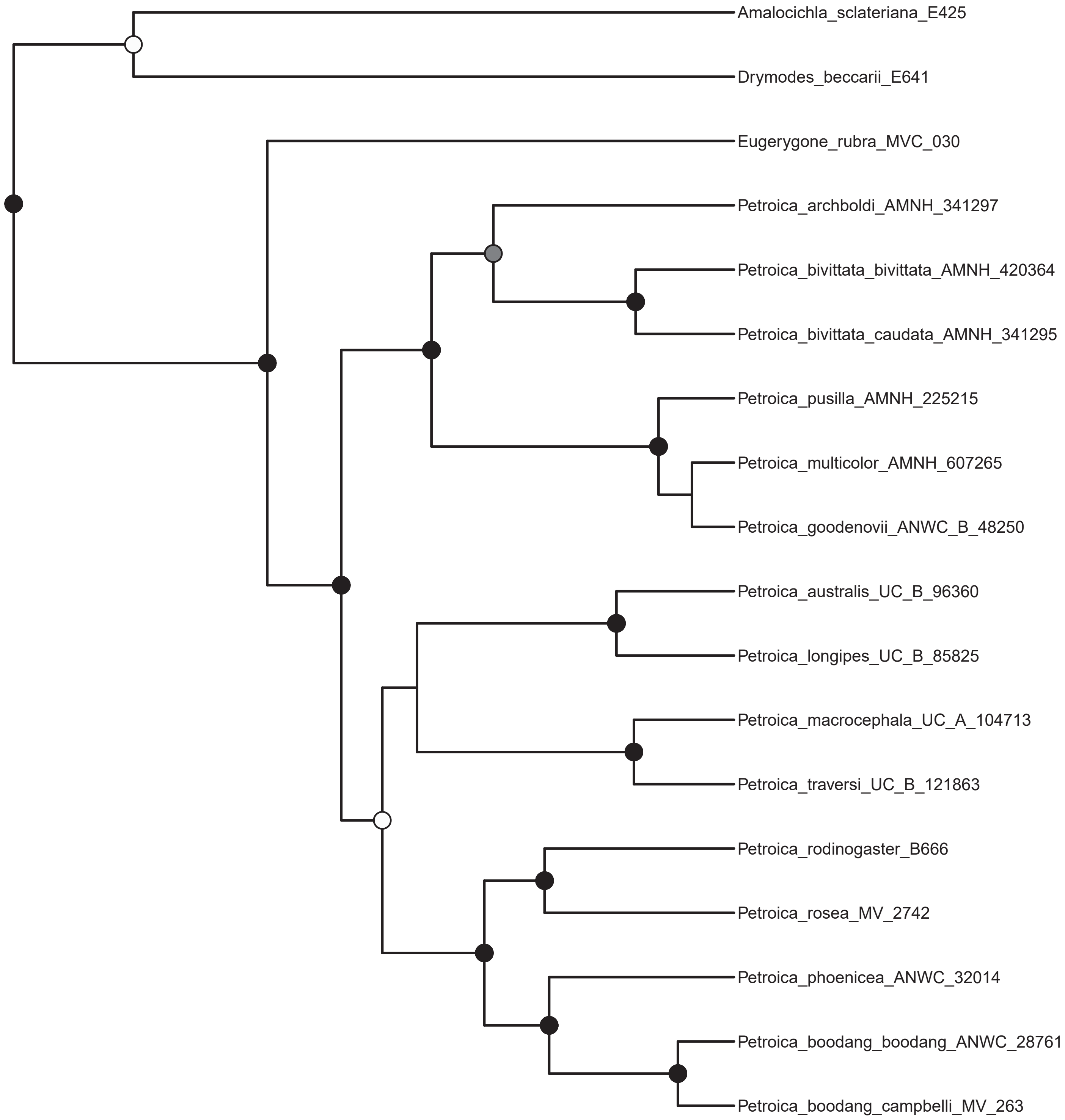
Amalocichla_sclateriana_E425
Drymodes_beccarii_E641
Eugerygone_rubra_MVC_030
Petroica_archboldi_AMNH_341297
Petroica_bivittata_bivittata_AMNH_420364
Petroica_bivittata_caudata_AMNH_341295
Petroica_pusilla_AMNH_225215
Petroica_multicolor_AMNH_607265
Petroica_goodenovii_ANWC_B_48250
Petroica_australis_UC_B_96360
Petroica_longipes_UC_B_85825
Petroica_macrocephala_UC_A_104713
Petroica_traversi_UC_B_121863
Petroica_rodinogaster_B666
Petroica_rosea_MV_2742
Petroica_phoenicea_ANWC_32014
Petroica_boodang_boodang_ANWC_28761
Petroica_boodang_campbelli_MV_263

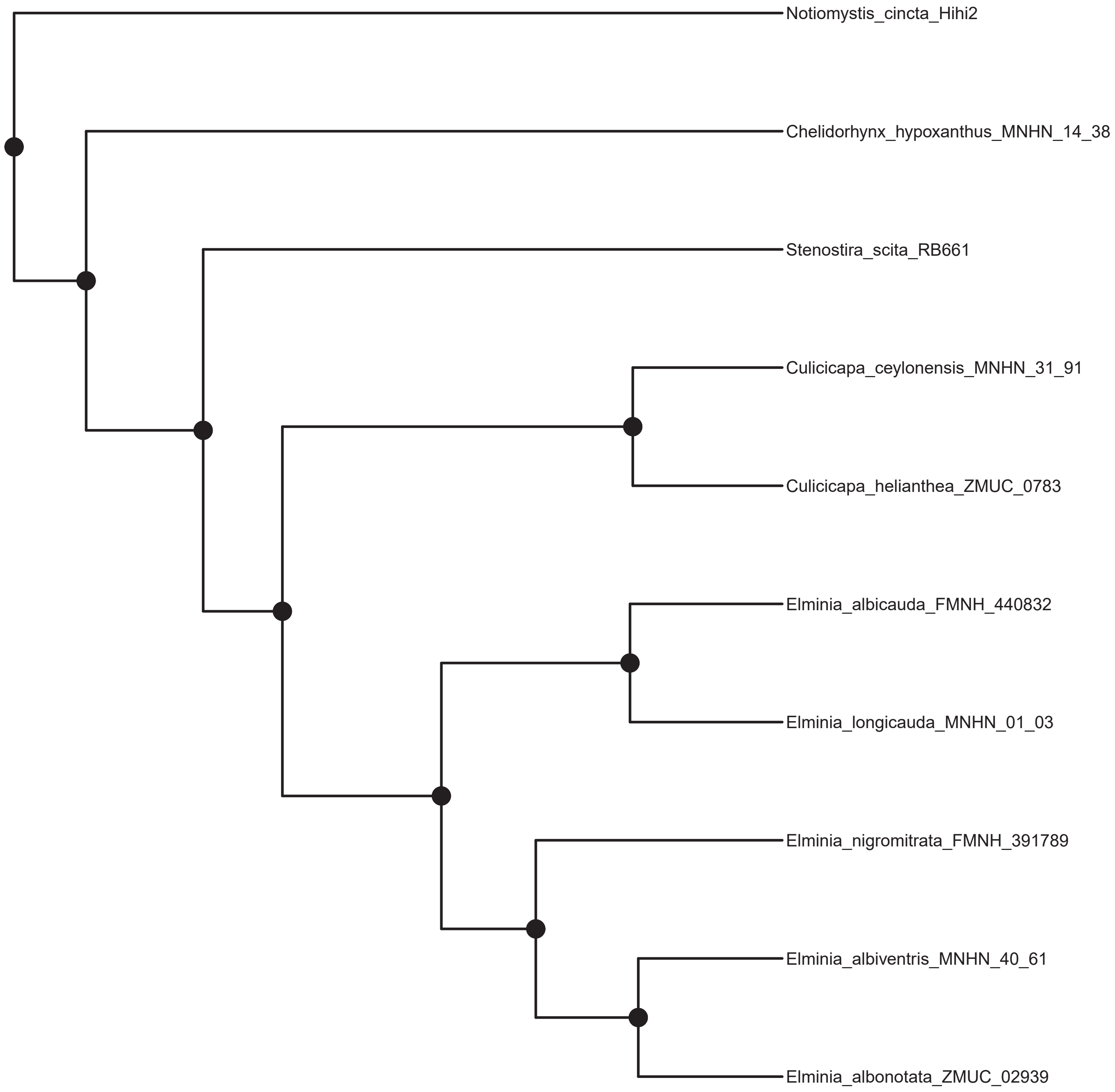
Notiomystis_cincta_Hihi2
Chelidorhynx_hypoxanthus_MNHN_14_38
Stenostira_scita_RB661
Culicicapa_ceylonensis_MNHN_31_91
Culicicapa_helianthea_ZMUC_0783
Elminia_albicauda_FMNH_440832
Elminia_longicauda_MNHN_01_03
Elminia_nigromitrata_FMNH_391789
Elminia_albiventris_MNHN_40_61
Elminia_albonotata_ZMUC_02939

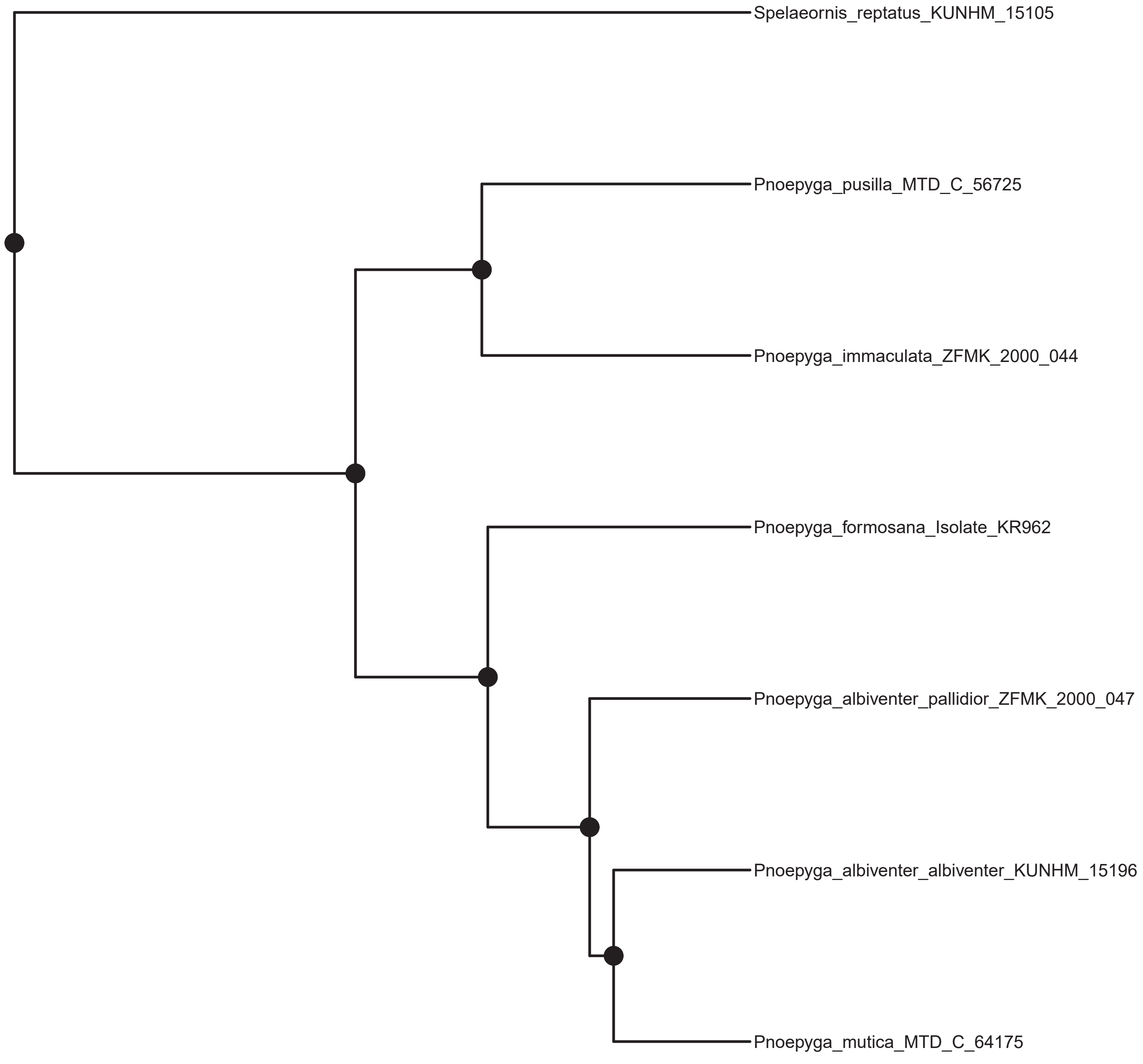
Spelaeornis_reptatus_KUNHM_15105
Pnoepyga_pusilla_MTD_C_56725
Pnoepyga_immaculata_ZFMK_2000_044
Pnoepyga_formosana_Isolate_KR962
Pnoepyga_albiventer_pallidior_ZFMK_2000_047
Pnoepyga_albiventer_albiventer_KUNHM_15196
Pnoepyga_mutica_MTD_C_64175

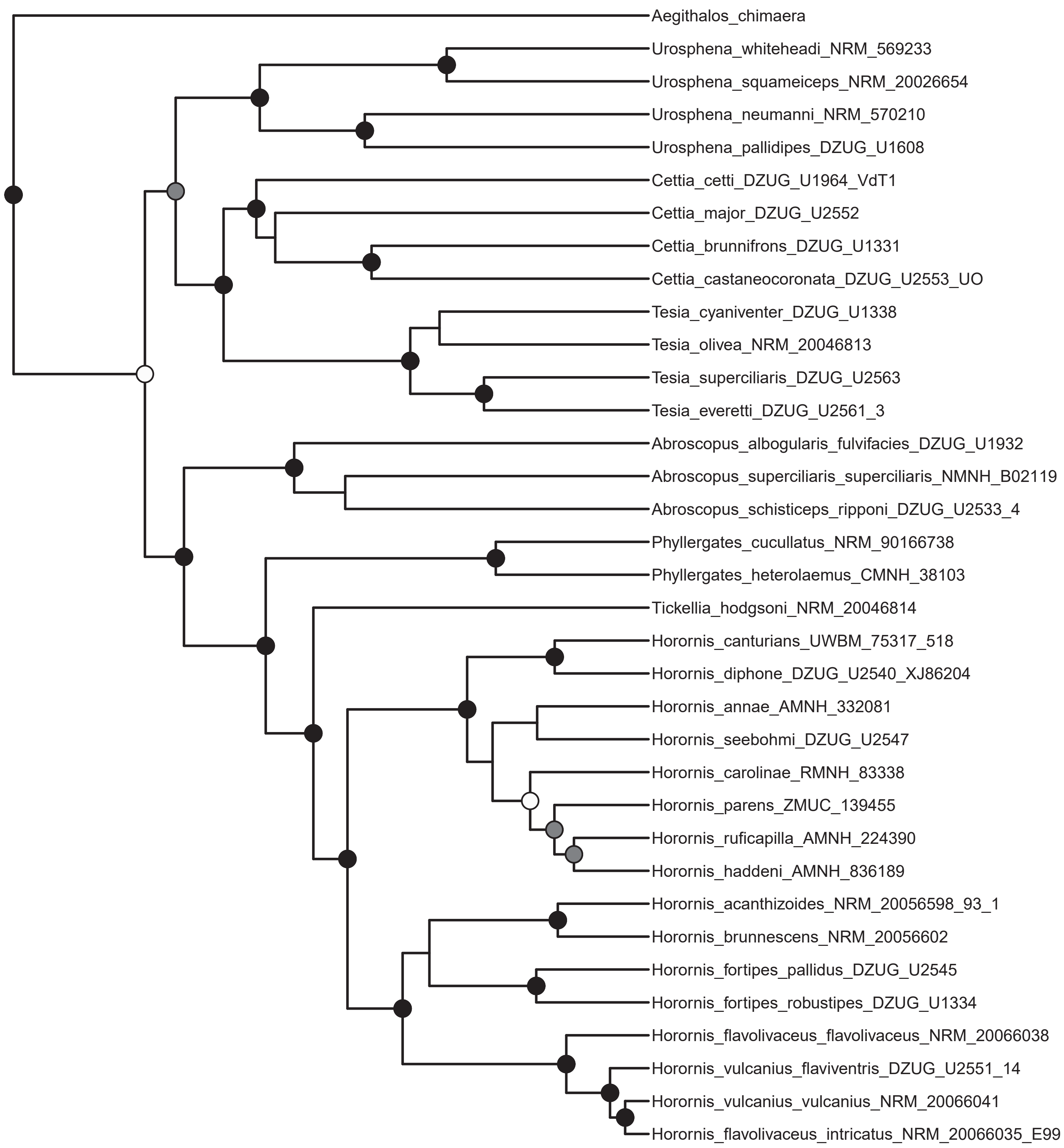
Aegithalos_chimaera
Urosphena_whiteheadi_NRM_569233
Urosphena_squameiceps_NRM_20026654
Urosphena_neumanni_NRM_570210
Urosphena_pallidipes_DZUG_U1608
Cettia_cetti_DZUG_U1964_VdT1
Cettia_major_DZUG_U2552
Cettia_brunnifrons_DZUG_U1331
Cettia_castaneocoronata_DZUG_U2553_UO
Tesia_cyaniventer_DZUG_U1338
Tesia_olivea_NRM_20046813
Tesia_superciliaris_DZUG_U2563
Tesia_everetti_DZUG_U2561_3
Abroscopus_albogularis_fulvifacies_DZUG_U1932
Abroscopus_superciliaris_superciliaris_NMNH_B02119
Abroscopus_schisticeps_ripponi_DZUG_U2533_4
Phyllergates_cucullatus_NRM_90166738
Phyllergates_heterolaemus_CMNH_38103
Tickellia_hodgsoni_NRM_20046814
Horornis_canturians_UWBM_75317_518
Horornis_diphone_DZUG_U2540_XJ86204
Horornis_annae_AMNH_332081
Horornis_seebohmi_DZUG_U2547
Horornis_carolinae_RMNH_83338
Horornis_parens_ZMUC_139455
Horornis_ruficapilla_AMNH_224390
Horornis_haddeni_AMNH_836189
Horornis_acanthizoides_NRM_20056598_93_1
Horornis_brunnescens_NRM_20056602
Horornis_fortipes_pallidus_DZUG_U2545
Horornis_fortipes_robustipes_DZUG_U1334
Horornis_flavolivaceus_flavolivaceus_NRM_20066038
Horornis_vulcanius_flaviventris_DZUG_U2551_14
Horornis_vulcanius_vulcanius_NRM_20066041
Horornis_flavolivaceus_intricatus_NRM_20066035_E99

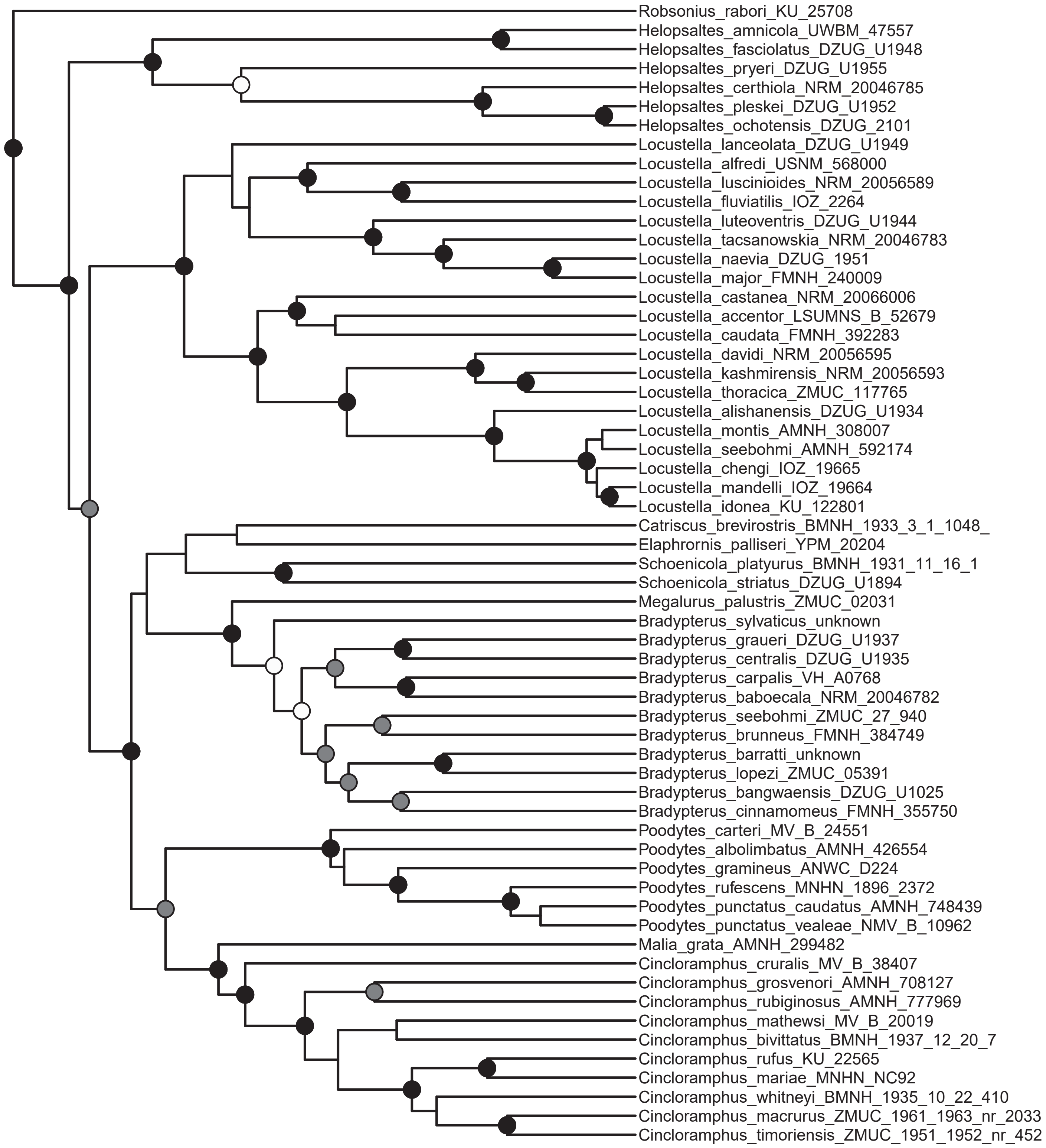
Robsonius_rabori_KU_25708
Helopsaltes_amnicola_UWBM_47557
Helopsaltes_fasciolatus_DZUG_U1948
Helopsaltes_pryeri_DZUG_U1955
Helopsaltes_certhiola_NRM_20046785
Helopsaltes_pleskei_DZUG_U1952
Helopsaltes_ochotensis_DZUG_2101
Locustella_lanceolata_DZUG_U1949
Locustella_alfredi_USNM_568000
Locustella_luscinioides_NRM_20056589
Locustella_fluviatilis_IOZ_2264
Locustella_luteoventris_DZUG_U1944
Locustella_tacsanowskia_NRM_20046783
Locustella_naevia_DZUG_1951
Locustella_major_FMNH_240009
Locustella_castanea_NRM_20066006
Locustella_accentor_LSUMNS_B_52679
Locustella_caudata_FMNH_392283
Locustella_davidi_NRM_20056595
Locustella_kashmirensis_NRM_20056593
Locustella_thoracica_ZMUC_117765
Locustella_alishanensis_DZUG_U1934
Locustella_montis_AMNH_308007
Locustella_seebohmi_AMNH_592174
Locustella_chengi_IOZ_19665
Locustella_mandelli_IOZ_19664
Locustella_idonea_KU_122801
Catriscus_brevirostris_BMNH_1933_3_1_1048_
Elaphrornis_palliseri_YPM_20204
Schoenicola_platyurus_BMNH_1931_11_16_1
Schoenicola_striatus_DZUG_U1894
Megalurus_palustris_ZMUC_02031
Bradypterus_sylvaticus_unknown
Bradypterus_graueri_DZUG_U1937
Bradypterus_centralis_DZUG_U1935
Bradypterus_carpalis_VH_A0768
Bradypterus_baboecala_NRM_20046782
Bradypterus_seebohmi_ZMUC_27_940
Bradypterus_brunneus_FMNH_384749
Bradypterus_barratti_unknown
Bradypterus_lopezi_ZMUC_05391
Bradypterus_bangwaensis_DZUG_U1025
Bradypterus_cinnamomeus_FMNH_355750
Poodytes_carteri_MV_B_24551
Poodytes_albolimbatus_AMNH_426554
Poodytes_gramineus_ANWC_D224
Poodytes_rufescens_MNHN_1896_2372
Poodytes_punctatus_caudatus_AMNH_748439
Poodytes_punctatus_vealeae_NMV_B_10962
Malia_grata_AMNH_299482
Cincloramphus_cruralis_MV_B_38407
Cincloramphus_grosvenori_AMNH_708127
Cincloramphus_rubiginosus_AMNH_777969
Cincloramphus_mathewsi_MV_B_20019
Cincloramphus_bivittatus_BMNH_1937_12_20_7
Cincloramphus_rufus_KU_22565
Cincloramphus_mariae_MNHN_NC92
Cincloramphus_whitneyi_BMNH_1935_10_22_410
Cincloramphus_macrurus_ZMUC_1961_1963_nr_2033
Cincloramphus_timoriensis_ZMUC_1951_1952_nr_452

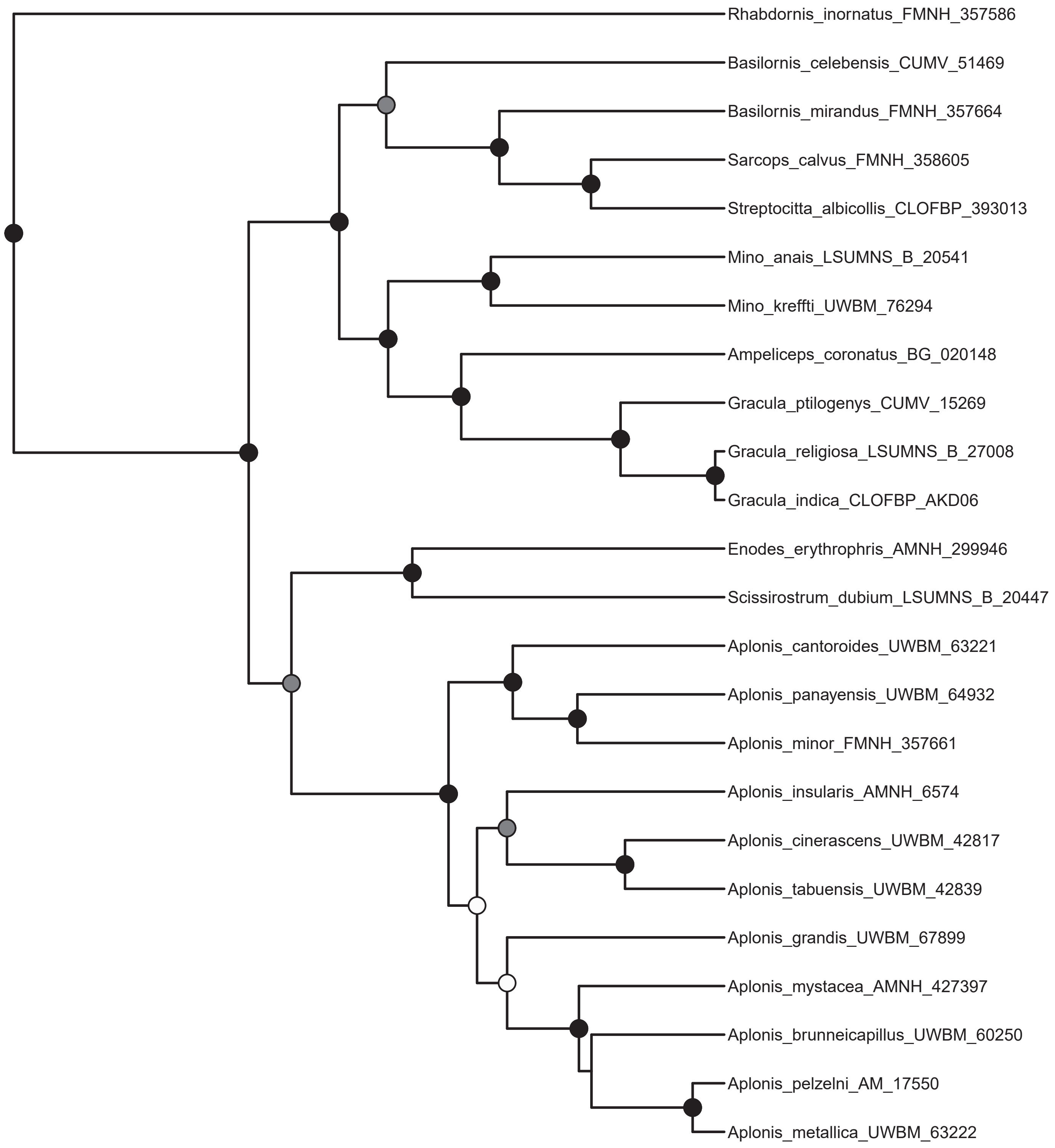
Rhabdornis_inornatus_FMNH_357586
Basilornis_celebensis_CUMV_51469
Basilornis_mirandus_FMNH_357664
Sarcops_calvus_FMNH_358605
Streptocitta_albicollis_CLOFBP_393013
Mino_anais_LSUMNS_B_20541
Mino_kreffti_UWBM_76294
Ampeliceps_coronatus_BG_020148
Gracula_ptilogenys_CUMV_15269
Gracula_religiosa_LSUMNS_B_27008
Gracula_indica_CLOFBP_AKD06
Enodes_erythrophris_AMNH_299946
Scissirostrum_dubium_LSUMNS_B_20447
Aplonis_cantoroides_UWBM_63221
Aplonis_panayensis_UWBM_64932
Aplonis_minor_FMNH_357661
Aplonis_insularis_AMNH_6574
Aplonis_cinerascens_UWBM_42817
Aplonis_tabuensis_UWBM_42839
Aplonis_grandis_UWBM_67899
Aplonis_mystacea_AMNH_427397
Aplonis_brunneicapillus_UWBM_60250
Aplonis_pelzelni_AM_17550
Aplonis_metallica_UWBM_63222

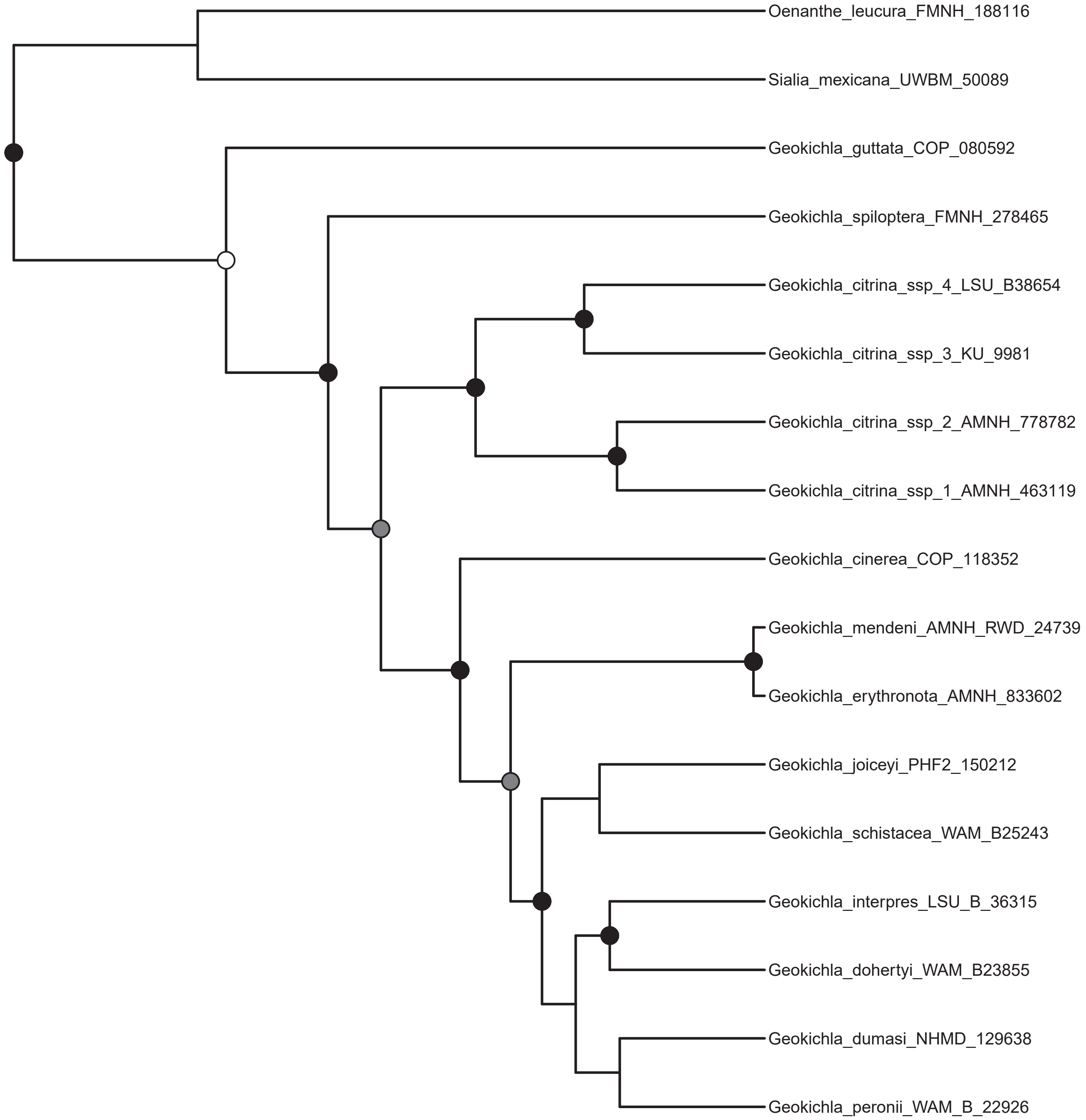

Oenanthe_leucura_FMNH_188116
Sialia_mexicana_UWBM_50089
Geokichla_guttata_COP_080592
Geokichla_spiloptera_FMNH_278465
Geokichla_citrina_ssp_4_LSU_B38654
Geokichla_citrina_ssp_3_KU_9981
Geokichla_citrina_ssp_2_AMNH_778782
Geokichla_citrina_ssp_1_AMNH_463119
Geokichla_cinerea_COP_118352
Geokichla_mendeni_AMNH_RWD_24739
Geokichla_erythronota_AMNH_833602
Geokichla_joiceyi_PHF2_150212
Geokichla_schistacea_WAM_B25243
Geokichla_interpres_LSU_B_36315
Geokichla_dohertyi_WAM_B23855
Geokichla_dumasi_NHMD_129638
Geokichla_peronii_WAM_B_22926

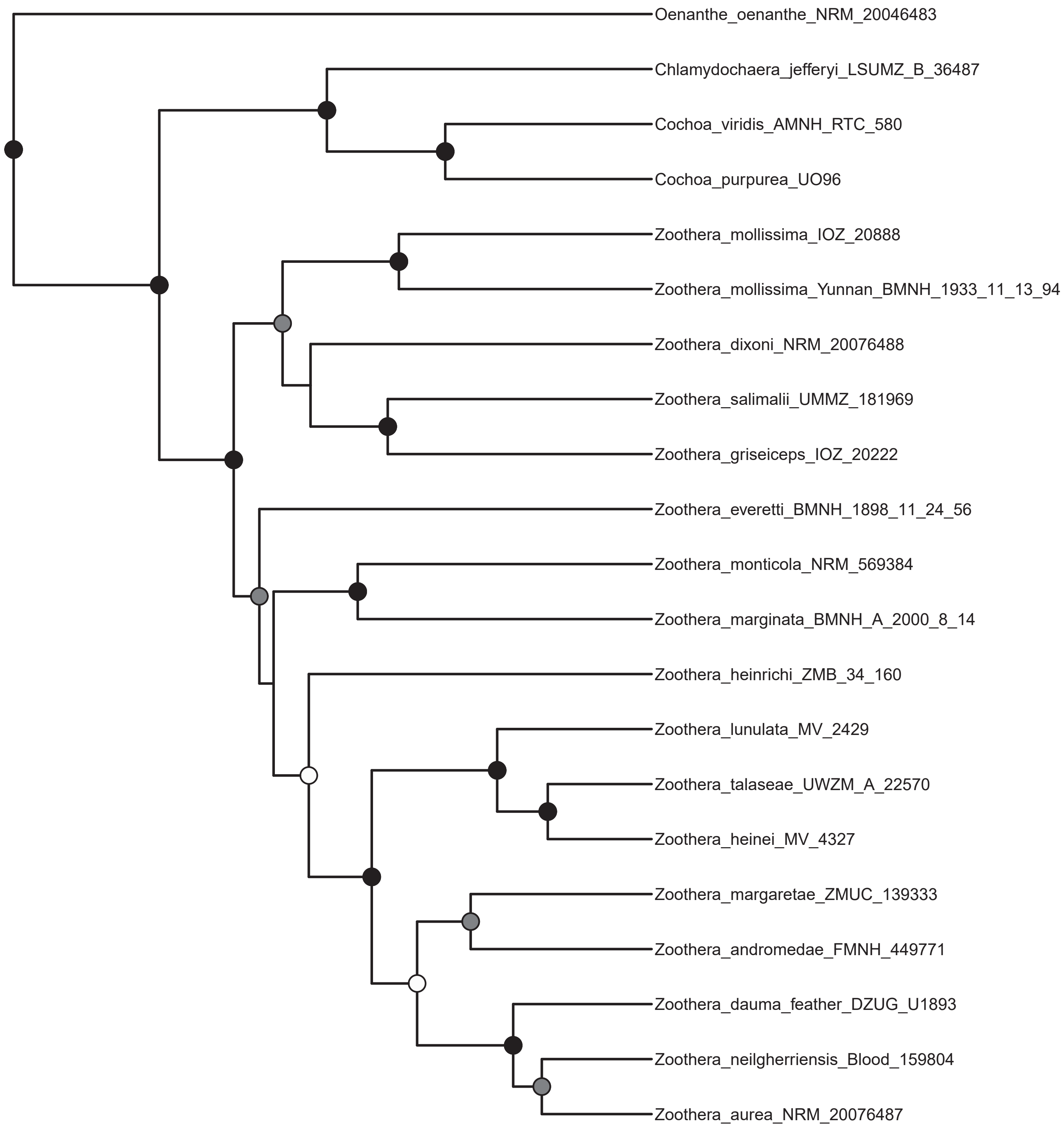
Oenanthe_oenanthe_NRM_20046483
Chlamydochaera_jefferyi_LSUMZ_B_36487
Cochoa_viridis_AMNH_RTC_580
Cochoa_purpurea_UO96
Zoothera_mollissima_IOZ_20888
Zoothera_mollissima_Yunnan_BMNH_1933_11_13_94
Zoothera_dixoni_NRM_20076488
Zoothera_salimalii_UMMZ_181969
Zoothera_griseiceps_IOZ_20222
Zoothera_everetti_BMNH_1898_11_24_56
Zoothera_monticola_NRM_569384
Zoothera_marginata_BMNH_A_2000_8_14
Zoothera_heinrichi_ZMB_34_160
Zoothera_lunulata_MV_2429
Zoothera_talaseae_UWZM_A_22570
Zoothera_heinei_MV_4327
Zoothera_margaretae_ZMUC_139333
Zoothera_andromedae_FMNH_449771
Zoothera_dauma_feather_DZUG_U1893
Zoothera_neilgherriensis_Blood_159804
Zoothera_aurea_NRM_20076487

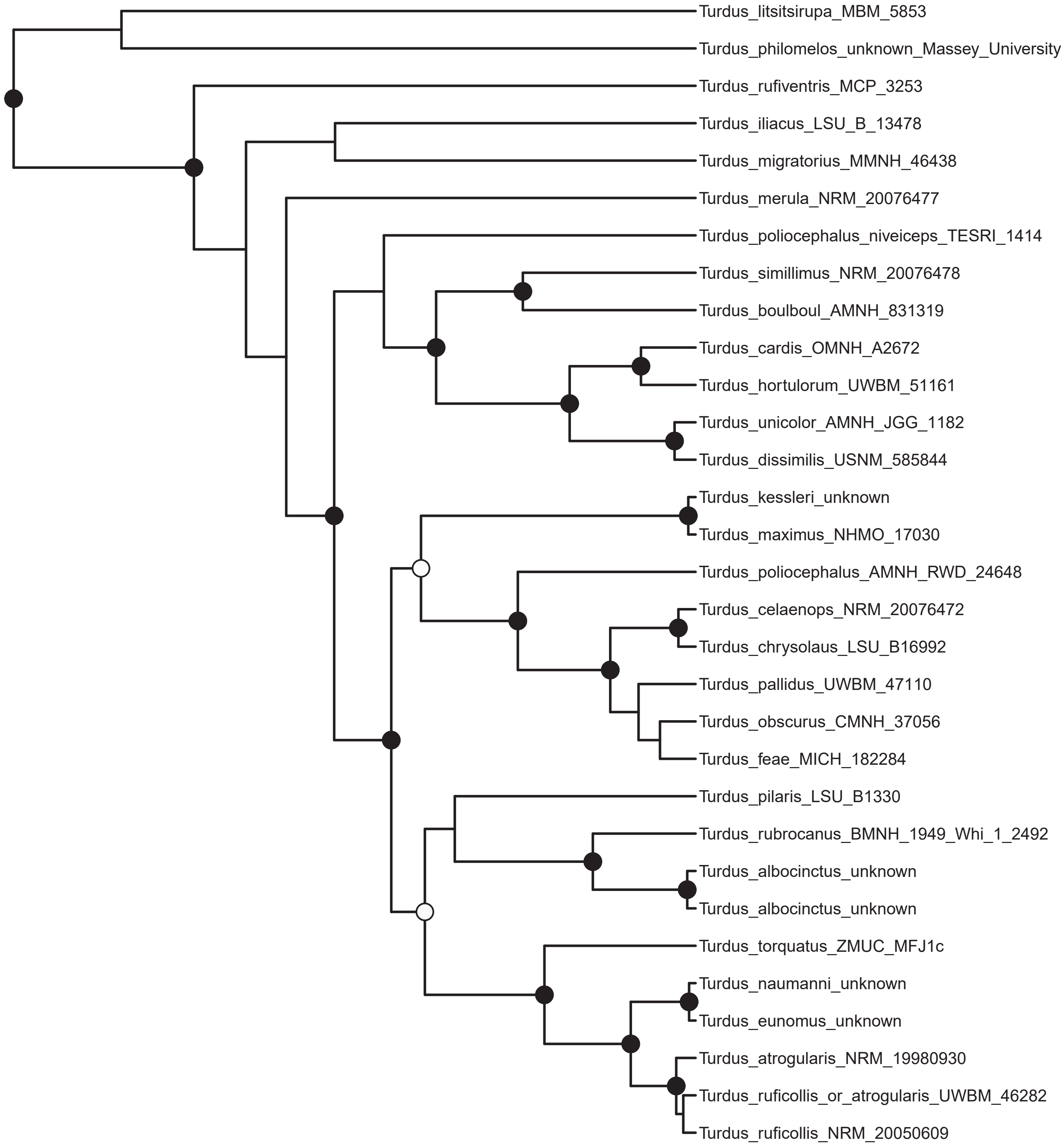
Turdus_litsitsirupa_MBM_5853
Turdus_philomelos_unknown_Massey_University
Turdus_rufiventris_MCP_3253
Turdus_iliacus_LSU_B_13478
Turdus_migratorius_MMNH_46438
Turdus_merula_NRM_20076477
Turdus_poliocephalus_niveiceps_TESRI_1414
Turdus_simillimus_NRM_20076478
Turdus_boulboul_AMNH_831319
Turdus_cardis_OMNH_A2672
Turdus_hortulorum_UWBM_51161
Turdus_unicolor_AMNH_JGG_1182
Turdus_dissimilis_USNM_585844
Turdus_kessleri_unknown
Turdus_maximus_NHMO_17030
Turdus_poliocephalus_AMNH_RWD_24648
Turdus_celaenops_NRM_20076472
Turdus_chrysolaus_LSU_B16992
Turdus_pallidus_UWBM_47110
Turdus_obscurus_CMNH_37056
Turdus_feae_MICH_182284
Turdus_pilaris_LSU_B1330
Turdus_rubrocanus_BMNH_1949_Whi_1_2492
Turdus_albocinctus_unknown
Turdus_albocinctus_unknown
Turdus_torquatus_ZMUC_MFJ1c
Turdus_naumanni_unknown
Turdus_eunomus_unknown
Turdus_atrogularis_NRM_19980930
Turdus_ruficollis_or_atrogularis_UWBM_46282
Turdus_ruficollis_NRM_20050609

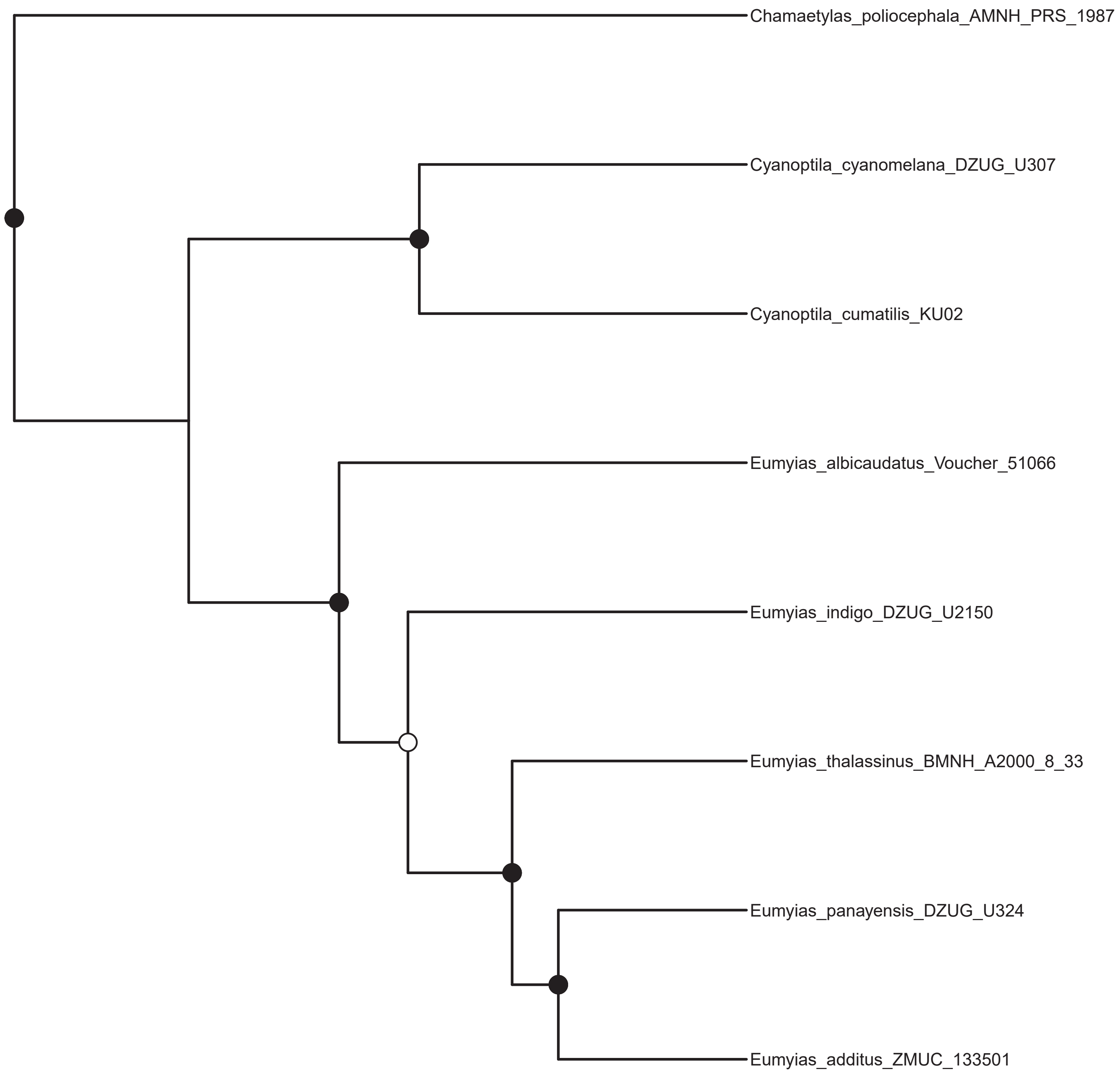
Chamaetylas_poliocephala_AMNH_PRS_1987
Cyanoptila_cyanomelana_DZUG_U307
Cyanoptila_cumatilis_KU02
Eumyias_albicaudatus_Voucher_51066
Eumyias_indigo_DZUG_U2150
Eumyias_thalassinus_BMNH_A2000_8_33
Eumyias_panayensis_DZUG_U324
Eumyias_additus_ZMUC_133501

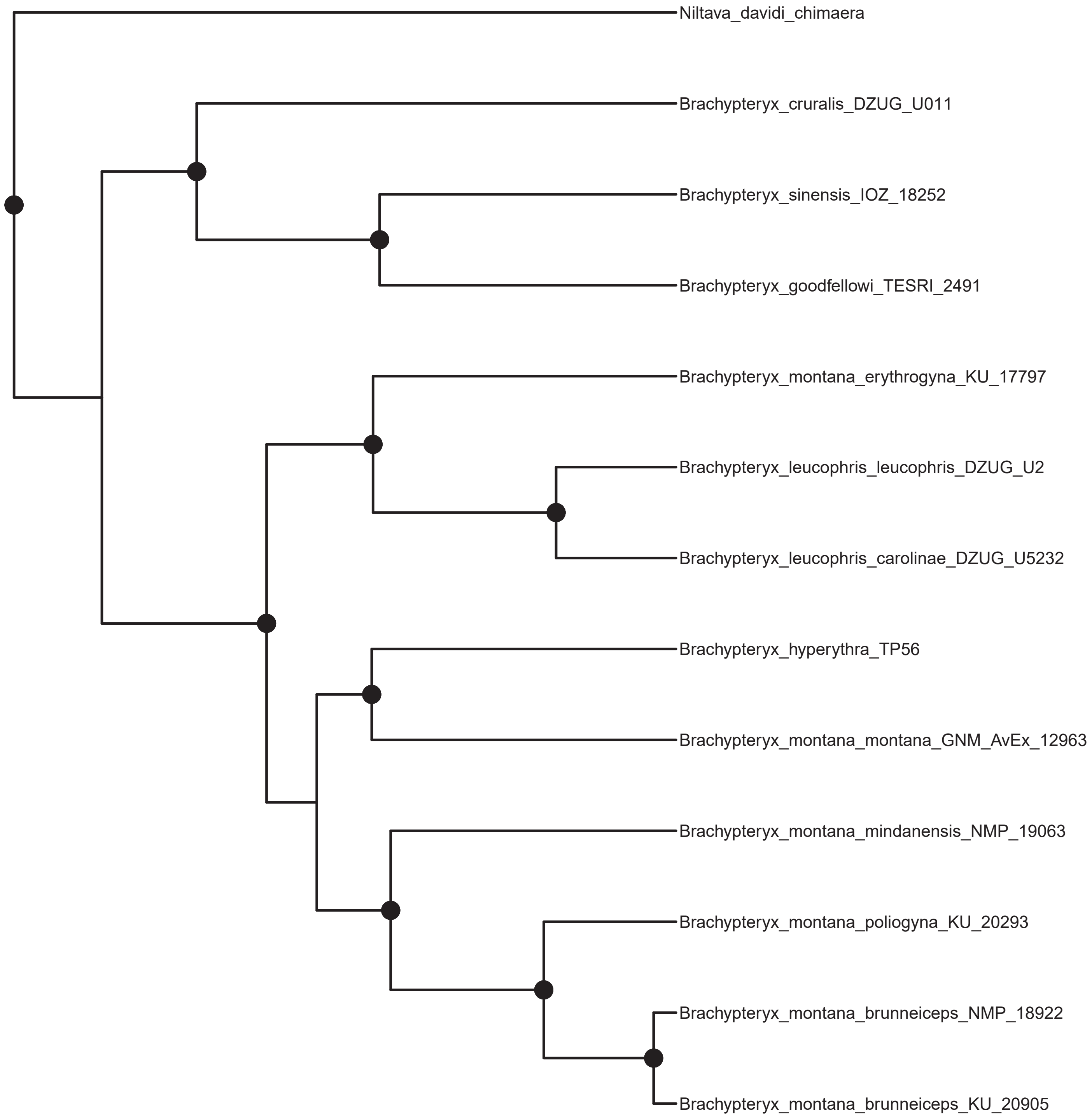
Niltava_davidi_chimaera
Brachypteryx_cruralis_DZUG_U011
Brachypteryx_sinensis_IOZ_18252
Brachypteryx_goodfellowi_TESRI_2491
Brachypteryx_montana_erythrogyna_KU_17797
Brachypteryx_leucophris_leucophris_DZUG_U2
Brachypteryx_leucophris_carolinae_DZUG_U5232
Brachypteryx_hyperythra_TP56
Brachypteryx_montana_montana_GNM_AvEx_12963
Brachypteryx_montana_mindanensis_NMP_19063
Brachypteryx_montana_poliogyna_KU_20293
Brachypteryx_montana_brunneiceps_NMP_18922
Brachypteryx_montana_brunneiceps_KU_20905

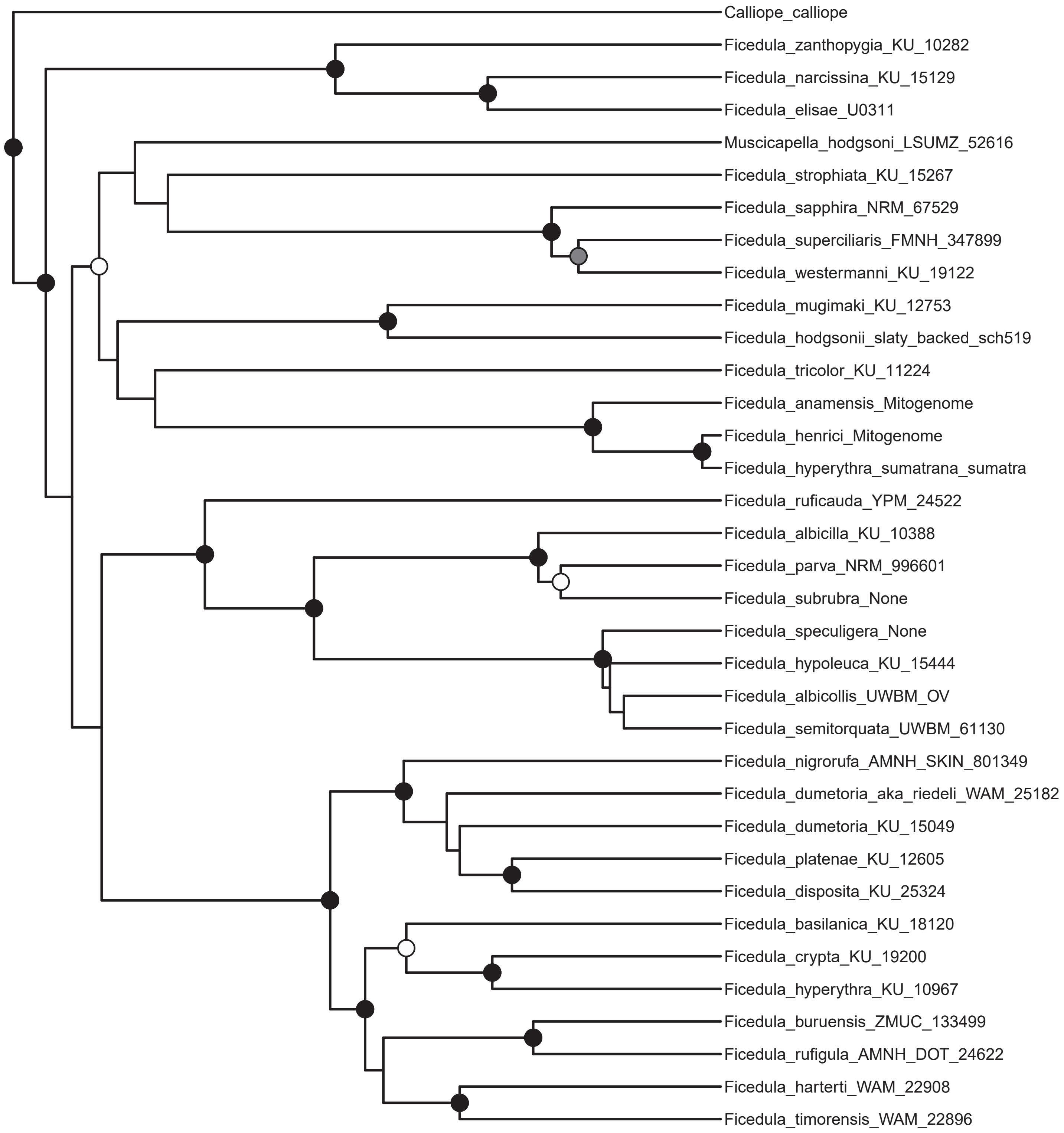
Calliope_calliope
Ficedula_zanthopygia_KU_10282
Ficedula_narcissina_KU_15129
Ficedula_elisae_U0311
Muscicapella_hodgsoni_LSUMZ_52616
Ficedula_strophiata_KU_15267
Ficedula_sapphira_NRM_67529
Ficedula_superciliaris_FMNH_347899
Ficedula_westermanni_KU_19122
Ficedula_mugimaki_KU_12753
Ficedula_hodgsonii_slaty_backed_sch519
Ficedula_tricolor_KU_11224
Ficedula_anamensis_Mitogenome
Ficedula_henrici_Mitogenome
Ficedula_hyperythra_sumatrana_sumatra
Ficedula_ruficauda_YPM_24522
Ficedula_albicilla_KU_10388
Ficedula_parva_NRM_996601
Ficedula_subrubra_None
Ficedula_speculigera_None
Ficedula_hypoleuca_KU_15444
Ficedula_albicollis_UWBM_OV
Ficedula_semitorquata_UWBM_61130
Ficedula_nigrorufa_AMNH_SKIN_801349
Ficedula_dumetoria_aka_riedeli_WAM_25182
Ficedula_dumetoria_KU_15049
Ficedula_platenae_KU_12605
Ficedula_disposita_KU_25324
Ficedula_basilanica_KU_18120
Ficedula_crypta_KU_19200
Ficedula_hyperythra_KU_10967
Ficedula_buruensis_ZMUC_133499
Ficedula_rufigula_AMNH_DOT_24622
Ficedula_harterti_WAM_22908
Ficedula_timorensis_WAM_22896

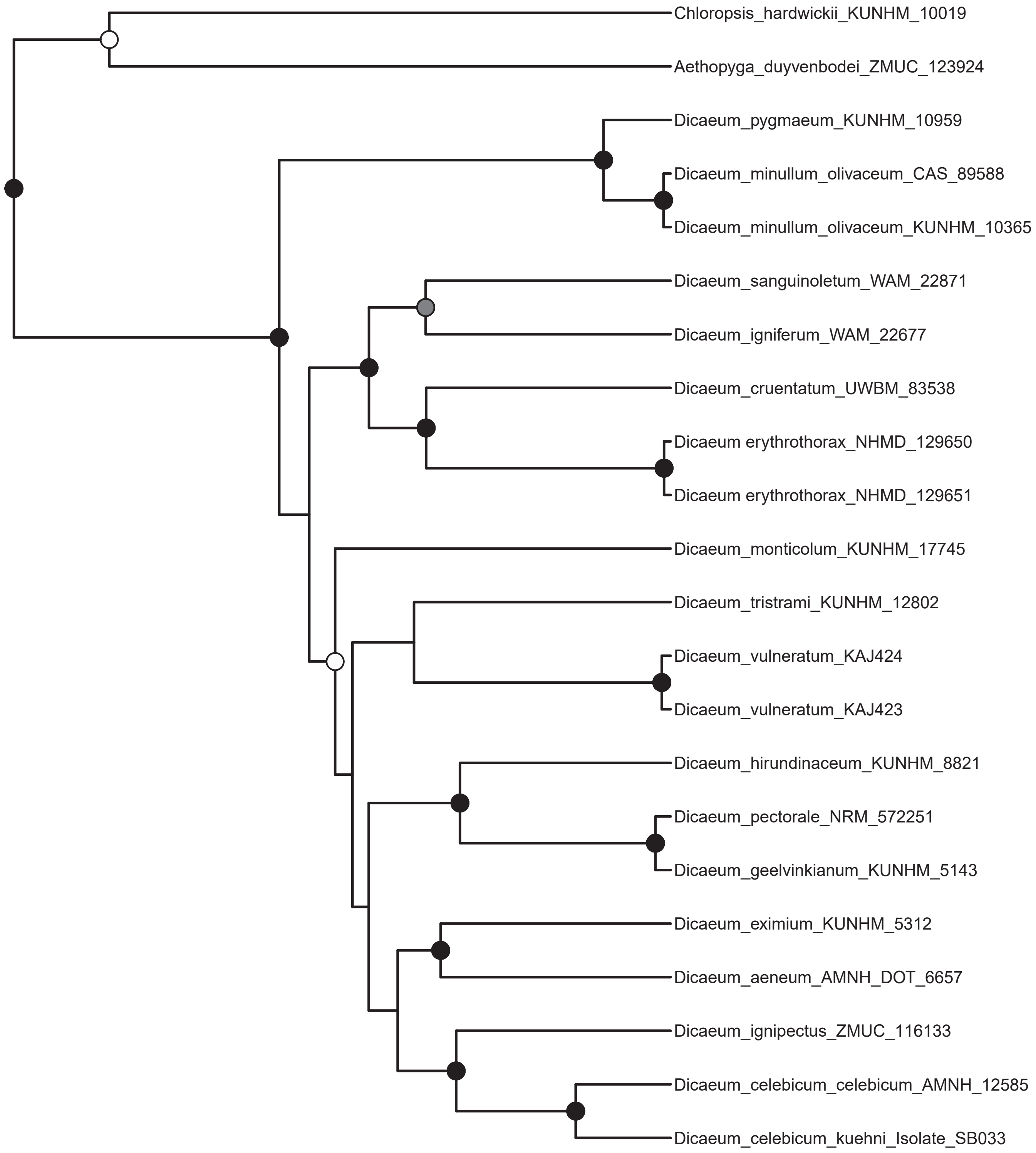
Chloropsis_hardwickii_KUNHM_10019
Aethopyga_duyvenbodei_ZMUC_123924
Dicaeum_pygmaeum_KUNHM_10959
Dicaeum_minullum_olivaceum_CAS_89588
Dicaeum_minullum_olivaceum_KUNHM_10365
Dicaeum_sanguinoletum_WAM_22871
Dicaeum_igniferum_WAM_22677
Dicaeum_cruentatum_UWBM_83538
Dicaeum erythrothorax_NHMD_129650
Dicaeum erythrothorax_NHMD_129651
Dicaeum_monticolum_KUNHM_17745
Dicaeum_tristrami_KUNHM_12802
Dicaeum_vulneratum_KAJ424
Dicaeum_vulneratum_KAJ423
Dicaeum_hirundinaceum_KUNHM_8821
Dicaeum_pectorale_NRM_572251
Dicaeum_geelvinkianum_KUNHM_5143
Dicaeum_eximium_KUNHM_5312
Dicaeum_aeneum_AMNH_DOT_6657
Dicaeum_ignipectus_ZMUC_116133
Dicaeum_celebicum_celebicum_AMNH_12585
Dicaeum_celebicum_kuehni_Isolate_SB033

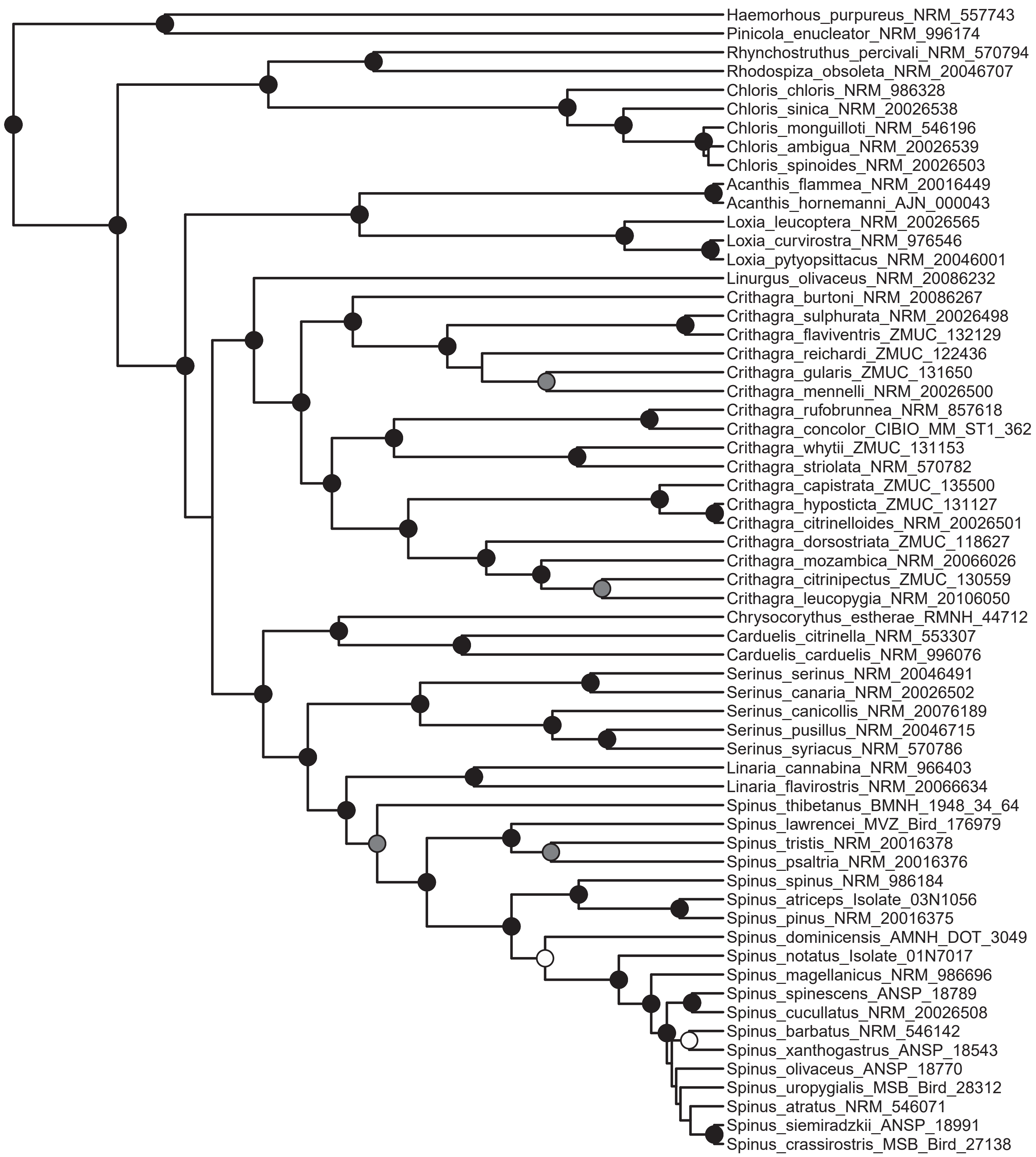
Haemorhous_purpureus_NRM_557743
Pinicola_enucleator_NRM_996174
Rhynchostruthus_percivali_NRM_570794
Rhodospiza_obsoleta_NRM_20046707
Chloris_chloris_NRM_986328
Chloris_sinica_NRM_20026538
Chloris_monguilloti_NRM_546196
Chloris_ambigua_NRM_20026539
Chloris_spinoides_NRM_20026503
Acanthis_flammea_NRM_20016449
Acanthis_hornemanni_AJN_000043
Loxia_leucoptera_NRM_20026565
Loxia_curvirostra_NRM_976546
Loxia_pytyopsittacus_NRM_20046001
Linurgus_olivaceus_NRM_20086232
Crithagra_burtoni_NRM_20086267
Crithagra_sulphurata_NRM_20026498
Crithagra_flaviventris_ZMUC_132129
Crithagra_reichardi_ZMUC_122436
Crithagra_gularis_ZMUC_131650
Crithagra_mennelli_NRM_20026500
Crithagra_rufobrunnea_NRM_857618
Crithagra_concolor_CIBIO_MM_ST1_362
Crithagra_whytii_ZMUC_131153
Crithagra_striolata_NRM_570782
Crithagra_capistrata_ZMUC_135500
Crithagra_hyposticta_ZMUC_131127
Crithagra_citrinelloides_NRM_20026501
Crithagra_dorsostriata_ZMUC_118627
Crithagra_mozambica_NRM_20066026
Crithagra_citrinipectus_ZMUC_130559
Crithagra_leucopygia_NRM_20106050
Chrysocorythus_estherae_RMNH_44712
Carduelis_citrinella_NRM_553307
Carduelis_carduelis_NRM_996076
Serinus_serinus_NRM_20046491
Serinus_canaria_NRM_20026502
Serinus_canicollis_NRM_20076189
Serinus_pusillus_NRM_20046715
Serinus_syriacus_NRM_570786
Linaria_cannabina_NRM_966403
Linaria_flavirostris_NRM_20066634
Spinus_thibetanus_BMNH_1948_34_64
Spinus_lawrencei_MVZ_Bird_176979
Spinus_tristis_NRM_20016378
Spinus_psaltria_NRM_20016376
Spinus_spinus_NRM_986184
Spinus_atriceps_Isolate_03N1056
Spinus_pinus_NRM_20016375
Spinus_dominicensis_AMNH_DOT_3049
Spinus_notatus_Isolate_01N7017
Spinus_magellanicus_NRM_986696
Spinus_spinescens_ANSP_18789
Spinus_cucullatus_NRM_20026508
Spinus_barbatus_NRM_546142
Spinus_xanthogastrus_ANSP_18543
Spinus_olivaceus_ANSP_18770
Spinus_uropygialis_MSB_Bird_28312
Spinus_atratus_NRM_546071
Spinus_siemiradzkii_ANSP_18991
Spinus_crassirostris_MSB_Bird_27138
