# Supplementary material for: The formation of the Indo-Pacific montane avifauna
Source: Nat Commun. 2023 Dec 11;14:8215. doi: 10.1038/s41467-023-43964-y (PMC10713610; doi:10.1038/s41467-023-43964-y)
Supplement: Supplementary file 6 — Supplementary Data 3 [file 41467_2023_43964_MOESM6_ESM.zip › Supplementary Data 3 Contents.pdf]

Supplementary Material for

The formation of the Indo-Pacific montane avifauna

Andrew Hart Reeve, Jonathan David Kennedy, Jose Martin Pujolar, Bent Petersen, Mozes P. K. Blom, Per Alström, Tri Haryoko, Per G. P. Ericson, Martin Irestedt, Johan A. A. Nylander, and Knud Andreas Jønsson

Supplementary Data 3. Tree files.

Contents:

1. Pachycephalidae tree for ancestral state reconstructions
2. Petroicidae A [*Microeca*] tree for ancestral state reconstructions
3. Petroicidae B [*Petroica*] tree for ancestral state reconstructions
4. Stenostiridae tree for ancestral state reconstructions
5. Pnoepygidae tree for ancestral state reconstructions
6. Cettiidae tree for ancestral state reconstructions
7. Phylloscopidae tree for ancestral state reconstructions (tree reused for the Indo-Pacific *Phylloscopus* leaf warblers supermatrix tree)
8. Locustellidae tree for ancestral state reconstructions
9. Sturnidae tree for ancestral state reconstructions
10. Turdidae A [*Geokichla*] tree for ancestral state reconstructions
11. Turdidae B [*Zoothera*] tree for ancestral state reconstructions
12. Turdidae C [*Turdus*] tree for ancestral state reconstructions
13. Muscicapidae A [*Eumyias*] tree for ancestral state reconstructions
14. Muscicapidae B [*Brachypteryx*] tree for ancestral state reconstructions
15. Muscicapidae C [*Ficedula*] tree for ancestral state reconstructions
16. Dicaeidae tree for ancestral state reconstructions
17. Fringillidae tree for ancestral state reconstructions
18. *Phyllergates cucullatus* supermatrix tree
19. *Phyllergates cucullatus* mitogenome tree
20. Indo-Pacific *Phylloscopus* leaf warblers mitogenome tree
21. *Ficedula hyperythra* supermatrix tree
22. *Ficedula hyperythra* mitogenome tree
23. 80-species tree used for phylogenetic null models
