# Supplementary material for: The formation of the Indo-Pacific montane avifauna
Source: Nat Commun. 2023 Dec 11;14:8215. doi: 10.1038/s41467-023-43964-y (PMC10713610; doi:10.1038/s41467-023-43964-y)
Supplement: Supplementary file 11 — Reporting Summary [file 41467_2023_43964_MOESM11_ESM.pdf]

nature portfolio

Corresponding author(s): Andrew Hart Reeve
Last updated by author(s): Nov 17, 2023

Reporting Summary

Nature Portfolio wishes to improve the reproducibility of the work that we publish. This form provides structure for consistency and transparency in reporting. For further information on Nature Portfolio policies, see our Editorial Policies and the Editorial Policy Checklist.

Statistics

For all statistical analyses, confirm that the following items are present in the figure legend, table legend, main text, or Methods section.

n/a	Confirmed	
☐	☒	The exact sample size (*n*) for each experimental group/condition, given as a discrete number and unit of measurement
☐	☒	A statement on whether measurements were taken from distinct samples or whether the same sample was measured repeatedly
☐	☒	The statistical test(s) used AND whether they are one- or two-sided<br>*Only common tests should be described solely by name; describe more complex techniques in the Methods section.*
☐	☒	A description of all covariates tested
☐	☒	A description of any assumptions or corrections, such as tests of normality and adjustment for multiple comparisons
☐	☒	A full description of the statistical parameters including central tendency (e.g. means) or other basic estimates (e.g. regression coefficient) AND variation (e.g. standard deviation) or associated estimates of uncertainty (e.g. confidence intervals)
☐	☒	For null hypothesis testing, the test statistic (e.g. *F*, *t*, *r*) with confidence intervals, effect sizes, degrees of freedom and *P* value noted<br>*Give P values as exact values whenever suitable.*
☐	☒	For Bayesian analysis, information on the choice of priors and Markov chain Monte Carlo settings
☒	☐	For hierarchical and complex designs, identification of the appropriate level for tests and full reporting of outcomes
☒	☐	Estimates of effect sizes (e.g. Cohen's *d*, Pearson's *r*), indicating how they were calculated

Our web collection on statistics for biologists contains articles on many of the points above.

Software and code

Policy information about availability of computer code

Data collection	We used the following for data collection (bioinformatics): ANGSD v.0.933, BWA v.0.7.12, FASTQC v.0.11.5, HMMer v.3.2.1, Megahit v.1.2.8, MITObim v.1.8, PEAR v.0.9.10, SAMtools v.0.1.19, Super Deduper v.1.4, and TRIMMOMATIC v.0.32. Illumina sequencing reads were cleaned using a custom-designed workflow to remove adapter contamination, low-quality bases and low-complexity reads (https://github.com/mozesblom/NGSdata_tools). Homologous regions corresponding to the multiple sequence alignments in the reference data were located and extracted from the genome assemblies using the BirdScanner workflow (https://github.com/Naturhistoriska/birdscanner).
Data analysis	We used the following for data analysis: ASTRAL III v.5.6.3, BEAST v.1.8.4, BioGeoBEARS v.1.1.2, BMGE v.1.12, geiger v.2.0, GNU parallel v.20210822, MAFFT v.7.310, motmot v.2.1.3, ParGenes v.1.0.1, phytools v.0.7-70, RAxML-NG v.0.9.0, SEAVIEW v.4.6.2, Tracer v.1.6, TreeAnnotator v.1.8.2, and TreeShrink v.1.3.3.

For manuscripts utilizing custom algorithms or software that are central to the research but not yet described in published literature, software must be made available to editors and reviewers. We strongly encourage code deposition in a community repository (e.g. GitHub). See the Nature Portfolio guidelines for submitting code & software for further information.

Data

Policy information about availability of data

All manuscripts must include a data availability statement. This statement should provide the following information, where applicable:

- Accession codes, unique identifiers, or web links for publicly available datasets
- A description of any restrictions on data availability
- For clinical datasets or third party data, please ensure that the statement adheres to our policy

Raw Illumina sequences are deposited in the Sequence Reads Archive, National Center for Biotechnology Information [https://www.ncbi.nlm.nih.gov/bioproject/PRJNA747888]. Individual gene sequences are deposited on GenBank, accession numbers OM991446–OM991474, ON015307–ON015422, and ON015448–ON015647. Additonal source data are presented in Supplementary Data 1-7. Source data for the Figures can be found in Supplementary Data 1 and 3.

Research involving human participants, their data, or biological material

Policy information about studies with human participants or human data. See also policy information about sex, gender (identity/presentation), and sexual orientation and race, ethnicity and racism.

Reporting on sex and gender	NA
Reporting on race, ethnicity, or other socially relevant groupings	NA
Population characteristics	NA
Recruitment	NA
Ethics oversight	NA

Note that full information on the approval of the study protocol must also be provided in the manuscript.

Field-specific reporting

Please select the one below that is the best fit for your research. If you are not sure, read the appropriate sections before making your selection.

☐ Life sciences ☐ Behavioural & social sciences ☒ Ecological, evolutionary & environmental sciences

For a reference copy of the document with all sections, see nature.com/documents/nr-reporting-summary-flat.pdf

Ecological, evolutionary & environmental sciences study design

All studies must disclose on these points even when the disclosure is negative.

Study description	The study is an investigation of the processes generating avian montane diversity using ancestral state reconstructions and population genomics. We used the following approach: 1. Identified all montane populations of passerine birds in two areas of the Indo-Pacific. 2. Performed phylogenetic analyses of these species and their relatives. 3. Performed ancestral state reconstructions using modern trait and distributional data. 4. Tested hypotheses pertaining to mountain colonization processes using modern and ancestral trait and distributional data, employing statistical tests including Mann-Whitney U-tests and chi-squared tests. 5. Tested for phylogenetic signal in the results from (4) using phylogenetic null models. 6. Performed genomic population studies of three species/clades with broad montane distributions in the focal region.
Research sample	We used genetic, distributional, and ecological data from Indo-Pacific passerine birds and their relatives, which provide a useful model for studying mountain colonization processes. We sampled with the goals of determining the evolutionary relationships of 1) all passerine species with montane populations in Wallacea and the Bismarcks/Solomons, and 2) individual populations of three species/clades with the broadest montane distributions across the focal islands. We supplemented newly generated genetic data with existing data from GenBank. All data sources are specified in the supplementary material accompanying the article.
Sampling strategy	We generated new genetic data from 69 individual birds, and sampled existing genetic data from an additional 525 individuals. Thorough geographic sampling allowed detailed population studies of three focal clades. Indo-Pacific passerine clades were sampled as comprehensively as possible given the breadth of existing data.
Data collection	A. H. Reeve and colleagues generated sequencing libraries. SciLifeLab performed sequencing. B. Petersen, M. P. K. Blom and colleagues performed bioinformatics. A. H. Reeve compiled distributional and ecological data. Data was collected and stored digitally.
Timing and spatial scale	Data were collected from 01/10/2017-01/12/2020. New genomic data were generated from birds occurring in the Asia-Pacific region. Existing genetic data were sampled from species from different regions across the globe.

Data exclusions	No data were excluded from the analyses.
Reproducibility	All data and custom code have been made available, and all methods are thoroughly described to ensure reproducibility. No experiments were conducted that required replication.
Randomization	We did not perform any experiments that would require randomization.
Blinding	The study design did not require blinding because there was no risk that observer bias would influence the results.

Did the study involve field work? ☐ Yes ☒ No

Reporting for specific materials, systems and methods

We require information from authors about some types of materials, experimental systems and methods used in many studies. Here, indicate whether each material, system or method listed is relevant to your study. If you are not sure if a list item applies to your research, read the appropriate section before selecting a response.

Materials & experimental systems

n/a	Involved in the study	
☒	☐	Antibodies
☒	☐	Eukaryotic cell lines
☒	☐	Palaeontology and archaeology
☐	☒	Animals and other organisms
☒	☐	Clinical data
☒	☐	Dual use research of concern
☒	☐	Plants

Methods

n/a	Involved in the study	
☒	☐	ChIP-seq
☒	☐	Flow cytometry
☒	☐	MRI-based neuroimaging

Animals and other research organisms

Policy information about studies involving animals; ARRIVE guidelines recommended for reporting animal research, and Sex and Gender in Research

Laboratory animals	NA
Wild animals	We sourced genetic samples from 69 individual passerine birds from various natural history museums. Species identifications and information on the provenance of all samples are provided in the supplementary material accompanying the article. The specific capture and euthanasia methods used for individual birds are not known.
Reporting on sex	Information on sex was not collected as it is irrelevant to the study.
Field-collected samples	DNA extraction and library preparation were performed at the Swedish Museum of Natural History. Samples were housed in freezers at the Department of Bioinformatics and Genetics.
Ethics oversight	No ethical approval or guidance was required for this study.

Note that full information on the approval of the study protocol must also be provided in the manuscript.

Plants

Seed stocks	NA
Novel plant genotypes	NA
Authentication	NA
